# Global beta-diversity of angiosperm trees is shaped by Quaternary climate change

**DOI:** 10.1126/sciadv.add8553

**Published:** 2023-04-05

**Authors:** Wu-Bing Xu, Wen-Yong Guo, Josep M. Serra-Diaz, Franziska Schrodt, Wolf L. Eiserhardt, Brian J. Enquist, Brian S. Maitner, Cory Merow, Cyrille Violle, Madhur Anand, Michaël Belluau, Hans Henrik Bruun, Chaeho Byun, Jane A. Catford, Bruno E. L. Cerabolini, Eduardo Chacón-Madrigal, Daniela Ciccarelli, J. Hans C. Cornelissen, Anh Tuan Dang-Le, Angel de Frutos, Arildo S. Dias, Aelton B. Giroldo, Alvaro G. Gutiérrez, Wesley Hattingh, Tianhua He, Peter Hietz, Nate Hough-Snee, Steven Jansen, Jens Kattge, Benjamin Komac, Nathan J. B. Kraft, Koen Kramer, Sandra Lavorel, Christopher H. Lusk, Adam R. Martin, Ke-Ping Ma, Maurizio Mencuccini, Sean T. Michaletz, Vanessa Minden, Akira S. Mori, Ülo Niinemets, Yusuke Onoda, Renske E. Onstein, Josep Peñuelas, Valério D. Pillar, Jan Pisek, Matthew J. Pound, Bjorn J. M. Robroek, Brandon Schamp, Martijn Slot, Miao Sun, Ênio E. Sosinski, Nadejda A. Soudzilovskaia, Nelson Thiffault, Peter M. van Bodegom, Fons van der Plas, Jingming Zheng, Jens-Christian Svenning, Alejandro Ordonez

**Affiliations:** ^1^ Center for Biodiversity Dynamics in a Changing World (BIOCHANGE), Department of Biology, Aarhus University, DK-8000 Aarhus C, Denmark.; ^2^ Section for Ecoinformatics and Biodiversity, Department of Biology, Aarhus University, DK-8000 Aarhus C, Denmark.; ^3^ German Centre for Integrative Biodiversity Research (iDiv) Halle-Jena-Leipzig, 04103 Leipzig, Germany.; ^4^ Zhejiang Tiantong Forest Ecosystem National Observation and Research Station and Research Center for Global Change and Complex Ecosystems, School of Ecological and Environmental Sciences, East China Normal University, 200241 Shanghai, P.R. China.; ^5^ Université de Lorraine, AgroParisTech, INRAE, Silva, Nancy, France.; ^6^ School of Geography, University of Nottingham, Nottingham, NG7 2RD, UK.; ^7^ Royal Botanic Gardens, Kew, Surrey TW9 3AE, UK.; ^8^ Department of Ecology and Evolutionary Biology, University of Arizona, Tucson, AZ 85721, USA.; ^9^ The Santa Fe Institute, 1399 Hyde Park Rd., Santa Fe, NM 87501, USA.; ^10^ Department of Ecology and Evolutionary Biology, University of Connecticut, Storrs, CT 06269, USA.; ^11^ CEFE, Univ Montpellier, CNRS, EPHE, IRD, Montpellier, France.; ^12^ School of Environmental Sciences, University of Guelph, Guelph, Ontario, Canada.; ^13^ Centre for Forest Research, Département des sciences biologiques, Université du Québec à Montréal, P.O. Box 8888, Centre-ville station, Montréal, QC H3C 3P8, Canada.; ^14^ Department of Biology, University of Copenhagen, 2100 Copenhagen Ø, Denmark.; ^15^ Department of Biological Sciences and Biotechnology, Andong National University, Andong, 36729, Korea.; ^16^ Department of Geography, King’s College London, London WC2B 4BG, UK.; ^17^ Department of Biotechnologies and Life Sciences, University of Insubria, Via Dunant 3, 21100 Varese, Italy.; ^18^ Herbario Luis Fournier Origgi, Centro de Investigación en Biodiversidad y Ecología Tropical (CIBET), Universidad de Costa Rica, San Pedro de Montes de Oca, 11501-2060 San José, Costa Rica.; ^19^ Department of Biology, University of Pisa, Via Luca Ghini 13, 56126 Pisa, Italy.; ^20^ Systems Ecology, A-LIFE, Faculty of Science, Vrije Universiteit, 1081 HV Amsterdam, Netherlands.; ^21^ Faculty of Biology - Biotechnology, University of Science - VNUHCM, 227 Nguyen Van Cu, District 5, 700000 Ho Chi Minh City, Vietnam.; ^22^ Goethe University, Institute for Physical Geography, Altenhöferallee 1, 60438 Frankfurt am Main, Germany.; ^23^ Departamento de Ensino, Instituto Federal de Educação, Ciências e Tecnologia do Ceará - IFCE campus Crateús, Avenida Geraldo Barbosa Marques, 567, 63708-260 Crateús, Brazil.; ^24^ Departamento de Ciencias Ambientales y Recursos Naturales Renovables, Facultad de Ciencias Agronómicas, Universidad de Chile, Santa Rosa 11315, La Pintana, Santiago, Chile.; ^25^ Institute of Ecology and Biodiversity (IEB), Santiago, Chile.; ^26^ Global Systems and Analytics, Nova Pioneer, Paulshof, Gauteng, South Africa.; ^27^ School of Molecular and Life Sciences, Curtin University, P.O. Box U1987, Perth, WA 6845, Australia.; ^28^ College of Science, Health, Engineering and Education, Murdoch University, Murdoch, WA, Australia.; ^29^ Institute of Botany, University of Natural Resources and Life Sciences Vienna, 1180 Vienna, Austria.; ^30^ Meadow Run Environmental, Leavenworth, WA 98826, USA.; ^31^ Institute of Systematic Botany and Ecology, Ulm University, 89081 Ulm, Germany.; ^32^ Max Planck Institute for Biogeochemistry, Hans Knöll Str. 10, 07745 Jena, Germany.; ^33^ Andorra Recerca + Innovació, AD600 Sant Julià de Lòria (Principat d'), Andorra.; ^34^ Department of Ecology and Evolutionary Biology, University of California, Los Angeles, Los Angeles, CA 90095, USA.; ^35^ Wageningen University, Forest Ecology and Management Group, Droevendaalsesteeg 4, 6700AA Wageningen, Netherlands.; ^36^ Land Life Company, Mauritskade 63, 1092AD, Amsterdam, Netherlands.; ^37^ Laboratoire d’Ecologie Alpine, LECA, UMR UGA-USMB-CNRS 5553, Université Grenoble Alpes, CS 40700, 38058 Grenoble Cedex 9, France.; ^38^ Environmental Research Institute, University of Waikato, Hamilton, New Zealand.; ^39^ Department of Physical and Environmental Sciences, University of Toronto Scarborough, 1265 Military Trail, M1C 1A4 Toronto, ON, Canada.; ^40^ State Key Laboratory of Vegetation and Environmental Change, Institute of Botany, Chinese Academy of Sciences, Beijing 100093, China.; ^41^ ICREA, Barcelona, 08010, Spain.; ^42^ CREAF, Cerdanyola del Vallès, Barcelona 08193, Catalonia, Spain.; ^43^ Department of Botany and Biodiversity Research Centre, University of British Columbia, Vancouver, BC V6T 1Z4, Canada.; ^44^ Department of Biology, Vrije Universiteit Brussel, Pleinlaan 2, 1050 Brussels, Belgium.; ^45^ Institute for Biology and Environmental Sciences, University of Oldenburg, 26129 Oldenburg, Germany.; ^46^ Research Center for Advanced Science and Technology, the University of Tokyo, 4-6-1 Komaba, Meguro, Tokyo 153-8904, Japan.; ^47^ Estonian University of Life Sciences, Kreutzwaldi 1, 51006 Tartu, Estonia.; ^48^ Division of Forest and Biomaterials Science, Graduate School of Agriculture, Kyoto University, Oiwake, Kitashirakawa, Kyoto, 606-8502 Japan.; ^49^ Naturalis Biodiversity Center, Darwinweg 2, 2333CR Leiden, Netherlands.; ^50^ CSIC, Global Ecology Unit CREAF- CSIC-UAB, Bellaterra, Barcelona 08193, Catalonia, Spain.; ^51^ Department of Ecology, Universidade Federal do Rio Grande do Sul, Porto Alegre, 91501-970, Brazil.; ^52^ Tartu Observatory, University of Tartu, Observatooriumi 1, Tõravere, 61602 Tartumaa, Estonia.; ^53^ Department of Geography and Environmental Sciences, Northumbria University, Newcastle upon Tyne NE1 8ST, UK.; ^54^ Aquatic Ecology and Environmental Biology, Faculty of Science, Radboud Institute for Biological and Environmental Sciences, Radboud University Nijmegen, 6525 AJ Nijmegen, Netherlands.; ^55^ Department of Biology, Algoma University, Sault Ste. Marie, Ontario, P6A 2G4, Canada.; ^56^ Smithsonian Tropical Research Institute, Apartado 0843-03092, Balboa, Ancón, Republic of Panama.; ^57^ National Key Laboratory for Germplasm Innovation & Utilization of Horticultural Crops, Huazhong Agricultural University, Wuhan 430070, China.; ^58^ Embrapa Clima Temperado, 96010-971 Pelotas, RS, Brazil.; ^59^ Centre for Environmental Sciences, Hasselt University, Martelarenlaan 42, 3500 Hasselt, Belgium.; ^60^ Natural Resources Canada, Canadian Wood Fibre Centre, 1055 du P.E.P.S., P.O. Box 10380, Stn. Sainte-Foy, Quebec, QC G1V 4C7, Canada.; ^61^ Institute of Environmental Sciences, Leiden University, 2333 CC Leiden, Netherlands.; ^62^ Plant Ecology and Nature Conservation Group, Wageningen University, Netherlands.; ^63^ Beijing Key Laboratory for Forest Resources and Ecosystem Processes, Beijing Forestry University, Beijing, 100083, China.; ^64^ Center for Ecological Dynamics in a Novel Biosphere (ECONOVO), Department of Biology, Aarhus University, DK-8000 Aarhus C, Denmark.

## Abstract

As Earth’s climate has varied strongly through geological time, studying the impacts of past climate change on biodiversity helps to understand the risks from future climate change. However, it remains unclear how paleoclimate shapes spatial variation in biodiversity. Here, we assessed the influence of Quaternary climate change on spatial dissimilarity in taxonomic, phylogenetic, and functional composition among neighboring 200-kilometer cells (beta-diversity) for angiosperm trees worldwide. We found that larger glacial-interglacial temperature change was strongly associated with lower spatial turnover (species replacements) and higher nestedness (richness changes) components of beta-diversity across all three biodiversity facets. Moreover, phylogenetic and functional turnover was lower and nestedness higher than random expectations based on taxonomic beta-diversity in regions that experienced large temperature change, reflecting phylogenetically and functionally selective processes in species replacement, extinction, and colonization during glacial-interglacial oscillations. Our results suggest that future human-driven climate change could cause local homogenization and reduction in taxonomic, phylogenetic, and functional diversity of angiosperm trees worldwide.

## Teaser

Quaternary climate change reduced and homogenized angiosperm tree diversity across large landscapes worldwide.

## INTRODUCTION

**Fig. 1.**
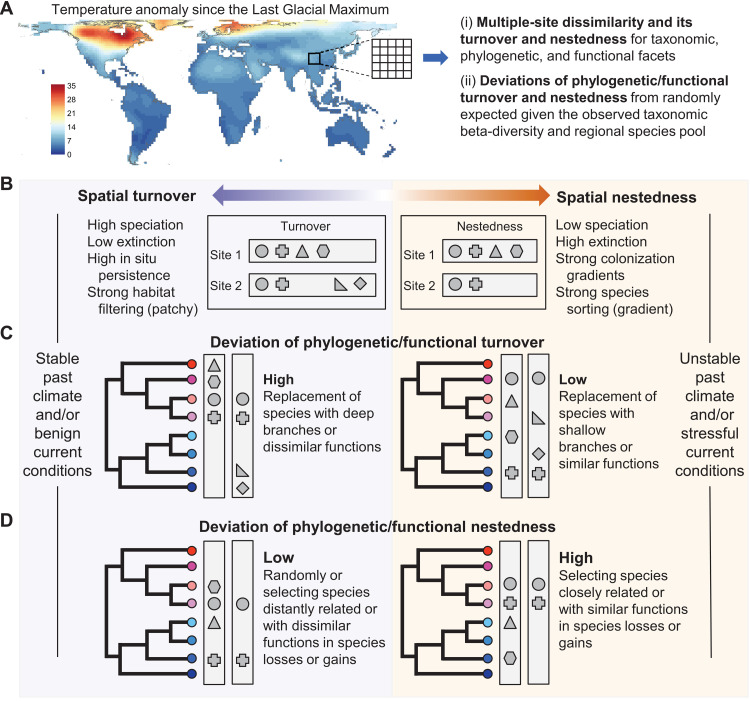
Hypothetical effects of past climatic stability and current environmental conditions on spatial turnover and nestedness components of taxonomic, phylogenetic, and functional beta-diversity. (**A**) Beta-diversity was measured as intraregional multiple-site dissimilarity within each moving window of 25 (5 × 5) grid cells of 200 km by 200 km worldwide. Total taxonomic, phylogenetic, and functional beta-diversity were decomposed into spatial turnover and nestedness components, respectively. Phylogenetic and functional turnover and nestedness were further compared with random expectations based on observed taxonomic beta-diversity and site-specific regional species pools and measured as deviations. The map shows the temperature anomaly since the LGM. (**B**) The turnover component is expected to be high in regions with stable past climate and/or benign current environmental conditions. In contrast, the nestedness component is expected to be high in regions with unstable past climate and/or stressful conditions, owing to both evolutionary and ecological processes. (**C** and **D**) Even if two regions have the same magnitudes of species turnover or nestedness, their phylogenetic and functional turnover or nestedness can be different because species replacements or losses/gains can occur among species that are closely related and similar in traits or distantly related and dissimilar in traits. In regions with unstable past climate and/or stressful conditions, deviations of phylogenetic and functional turnover from random expectations based on taxonomic beta-diversity are expected to be low if the replaced species come from closely related young lineages (shallow branches) and have similar trait values (colors) (**C**), and deviations of phylogenetic and functional nestedness are expected to be high if species losses/gains are phylogenetically and functionally selective, targeting closely related species and affiliated specific trait values (**D**). For clarity, only two sites are illustrated in each example region. Geometric shapes represent species and colors of dots functional trait values.

One of the main challenges for ecology in the Anthropocene is to understand how ongoing and near-future climate change reshapes the distribution of biodiversity and ecosystem functioning (*1*, *2*). As Earth’s climate has varied strongly through geological time, studying the impacts of past climate change on current biodiversity patterns provides an opportunity to understand the risks emerging from ongoing anthropogenic climate change (*3*–*5*). Notably, Earth has experienced major glaciations over the past 2.6 million years (the Quaternary), which have left lasting imprints on many organismal groups, including trees, through effects on speciation, extinction, and range shifts [as reviewed in (*6*)]. Although an increasing number of studies have assessed the impacts of paleoclimate change on biodiversity, most of these studies focused on patterns of taxonomic diversity of local assemblages (alpha-diversity) [e.g., (*7*–*10*)]. Biodiversity, however, includes other complementary dimensions, namely, phylogenetic and functional diversity, and also encompasses variation in assemblage composition across sites (beta-diversity) (*11*–*14*). Taxonomic, phylogenetic, and functional beta-diversity provide information on how compositions of species identities, evolutionary history, and functional traits vary across spatial gradients (Fig. 1). There is limited knowledge about the impacts of past rapid climate change on spatial patterns of these multiple facets of beta-diversity at large scales (*5*, *13*–*15*). Examining and comparing paleoclimatic legacies on taxonomic, phylogenetic, and functional beta-diversity are crucial for understanding the effects of ongoing climate change on future biodiversity patterns.

In previous studies, taxonomic beta-diversity was usually decomposed into two additive components: spatial species turnover and nestedness of assemblages (*16*). Variation in these two components is considered to be the consequence of different ecological and evolutionary processes (*16*–*20*). Spatial species turnover reflects species replacement between sites, while nestedness shows how depauperate assemblages are subsets of richer ones, reflecting species losses or gains across sites (*16*). Compared to regions that experienced unstable climates over glacial-interglacial cycles, regions with a relatively stable climate (usually low-latitude areas) have many more species persisting in situ and experience lower rates of species extinction and higher rates of speciation (*21*), which has led to more species with small ranges (*22*–*25*). As a result, spatial species turnover is expected to be the dominant component of beta-diversity in regions with stable past climates (Fig. 1B). By comparison, regions with unstable past climates experience higher local extinctions during climatically harsh periods such as glaciations, and many extant species are colonizers from glacial refugia (*5*, *21*). Because species’ postglacial recolonization may lag the emergence of suitable climatic conditions (*26*–*28*), some sites, particularly those far from the glacial refugia, are likely to lose more species than other sites during range expansion and contraction through glacial-interglacial cycles, leading to patterns of nestedness in assemblage compositions (Fig. 1B) (*17*, *19*, *29*).

Alternatively, spatial patterns of both turnover and nestedness components of beta-diversity may be shaped by contemporary environmental conditions (Fig. 1B) (*17*, *18*, *29*, *30*). In tropical areas with warm and wet conditions, a large number of species have accumulated, and these species are generally specialized with small range size favored by low climatic seasonality (*31*), leading to a high spatial species turnover (*13*, *32*). Topographic heterogeneity may also drive high species turnover through geographical isolation limiting dispersal (*12*). In addition, environmental heterogeneity, including topography-driven heterogeneity, can drive species turnover by selecting species with different environmental requirements at each local site (termed “habitat filtering”) (*18*, *33*, *34*). This filtering would be more apparent when species have narrow niche breadths [usually in tropical regions (*35*)]. Alternatively, environmental gradients can drive the nestedness structure of assemblages by gradually filtering out species by tolerance limits along gradients of increasingly stressful conditions (termed “species sorting”; Fig. 1B) (*33*, *34*). Furthermore, human pressures can decrease species turnover by reducing the distributions of small-ranged species and expanding the distributions of large-ranged species (*36*). They may also increase the nestedness of assemblages when a high proportion of species is lost due to habitat conversion in a subset of sites within a region.

There is some evidence for the paleoclimatic legacies on continental and global patterns of both turnover and nestedness components of taxonomic beta-diversity in multiple organismal groups, e.g., amphibians, freshwater fish, mammals, birds, and plants (*14*, *15*, *17*–*19*, *29*, *37*). However, only a few studies have simultaneously assessed paleoclimatic legacies on large-scale patterns of taxonomic, phylogenetic, and functional beta-diversity (*14*, *15*, *37*–*39*), particularly for megadiverse organismal groups, such as trees. Specifically, it remains unclear whether phylogenetic and functional beta-diversity have been influenced by paleoclimate change through processes beyond those affecting taxonomic beta-diversity. Like taxonomic beta-diversity, phylogenetic and functional beta-diversity can be decomposed into turnover and nestedness components. Phylogenetic and functional turnover reflects the replacement of evolutionary lineages and traits, and the nestedness captures losses/gains in lineages and functions across sites (*14*, *15*, *37*–*41*). Because species replacements or losses/gains among assemblages can occur among species that are either closely related and have similar traits or are distantly related and dissimilar in traits (*40*, *41*), regions with the same magnitude of taxonomic turnover or nestedness can have different phylogenetic and functional turnover or nestedness (Fig. 1, C and D). If species replacements or losses/gains are phylogenetically and functionally selective, then species that are replaced or lost/gained would be more closely (or distantly) related and similar (or dissimilar) in functional traits compared to those expected based on random processes. In this study, we propose a null model to calculate random expectations of phylogenetic and functional turnover and nestedness (i.e., expectations when a random group of species are replaced or lost/gained) for each given region based on its observed taxonomic beta-diversity and species pool (see the “Null model” section in Materials and Methods for details). By comparing observed and randomly expected beta-diversity, we can reveal whether phylogenetically and functionally selective processes are involved in species replacements, losses, and gains, which cumulatively shape phylogenetic and functional turnover and nestedness among assemblages (Fig. 1).

Past climatic stability can drive phylogenetic and functional turnover and nestedness to deviate from those randomly expected given taxonomic beta-diversity (Fig. 1, C and D). Regions with stable climates over glacial-interglacial cycles are usually the centers of paleoendemic species (*42*, *43*), which would lead to higher phylogenetic turnover than randomly expected from taxonomic beta-diversity. In contrast, few and relatively young endemic species are expected to be distributed in regions with unstable past climates (*43*, *44*), which would result in lower phylogenetic turnover than random expectations (Fig. 1C). These neoendemic species are expected to have similar functional trait values if trait divergence is slow (*45*). Therefore, regions with unstable past climates may have lower functional turnover than random expectations, particularly when traits are phylogenetically conserved. However, because certain traits may not necessarily be phylogenetically conserved, functional turnover may show a different pattern than phylogenetic turnover. At the same time, because certain functional traits, e.g., specific leaf area in plants, are closely associated with species adaptation to environmental conditions (*46*), deviations of functional turnover from random expectations may also be strongly influenced by contemporary environmental conditions. For example, species from different assemblages in stressful conditions tend to share similar functional trait values that can improve plant performance under local environmental conditions (*47*). By comparison, regions with unstable past climates may have higher phylogenetic and functional nestedness than random expectations (Fig. 1D). This pattern can emerge when glaciation-driven extinction and postglacial colonization are phylogenetically and functionally selective, targeting species that are closely related and affiliated with specific functional trait values (*48*, *49*). For instance, extinctions of temperate trees during glaciation periods are disproportionately common among cold-intolerant species (*48*, *50*), while species’ dispersal capacity has been shown to determine the extent to which they recolonize their climatically suitable areas after glaciations (*27*). These and other phylogenetically and functionally selective processes should drive a larger difference in phylogenetic and functional diversity (i.e., stronger nestedness structure) between species-rich and species-poor assemblages than those expected based on random species gain and loss processes (Fig. 1D).

While both past climate stability and contemporary environmental conditions may shape taxonomic, phylogenetic, and functional turnover and nestedness, whether and how these processes operate and their relative importance are poorly understood. No studies have so far simultaneously explored global patterns of these three facets of beta-diversity and assessed if phylogenetic and functional beta-diversity have been influenced by paleoclimate change through processes beyond those affecting taxonomic beta-diversity. Because trees and tree diversity play crucial roles in terrestrial ecosystems, global biodiversity, and people (*51*, *52*), it is particularly important to clarify global patterns of tree beta-diversity and their drivers. Here, we combined the most extensive global database of angiosperm tree species’ distributions (*53*) with information about their phylogenetic relationships and functional traits to quantify intraregional compositional dissimilarity patterns within moving windows of 25 (5 × 5) grid cells of 200 km by 200 km. We used Sørensen-based multiple-site dissimilarity to calculate the total beta-diversity and partition it into components of turnover (Simpson-based dissimilarity) and nestedness (difference between Sørensen and Simpson dissimilarities) for all three biodiversity facets (*16*, *54*). We then used a null model to calculate the deviations of observed phylogenetic and functional turnover and nestedness from random expectations given the observed taxonomic beta-diversity and site-specific regional species pools. Last, we used differences in annual temperature and precipitation between the present and the Last Glacial Maximum (LGM; ~21,000 years ago) (temperature and precipitation anomalies) to represent past climate stability and current climatic, topographic, and human pressure variables to represent contemporary environmental conditions and ultimately assessed their relative roles in shaping present patterns of beta-diversity in angiosperm trees.

We hypothesize that both past climate stability and contemporary environmental conditions affect spatial patterns of both turnover and nestedness components of beta-diversity across three biodiversity facets, as illustrated in Fig. 1. We expect a strong influence of temperature anomaly since the LGM because of the importance of temperature for species distributions and the large temperature change during glacial-interglacial oscillations. We also hypothesize that phylogenetic and functional turnover would be lower, and the nestedness would be higher than randomly expected from taxonomic beta-diversity in regions with unstable past climates as a result of phylogenetically and functionally selective processes in species replacement, extinction, and colonization during glacial-interglacial oscillations (Fig. 1, C and D).

## RESULTS

### Global patterns of taxonomic, phylogenetic, and functional beta-diversity in angiosperm trees

**Fig. 2.**
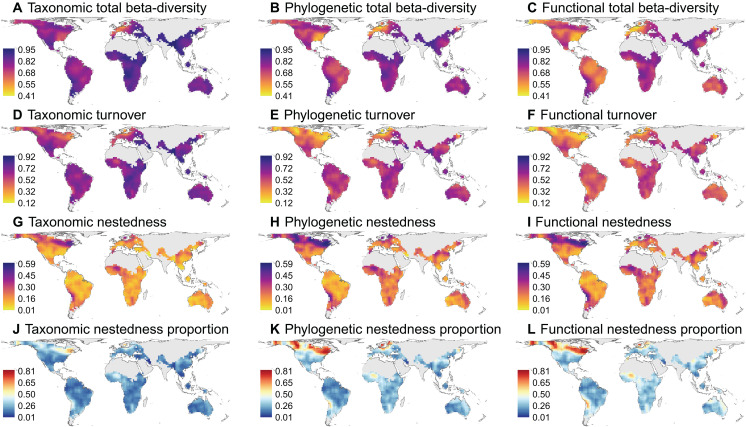
Global patterns of taxonomic, phylogenetic, and functional beta-diversity of angiosperm trees. Total beta-diversity (**A** to **C**) and its components of turnover (**D** to **F**) and nestedness (**G** to **I**) and the proportion of total beta-diversity contributed by nestedness (**J** to **L**) are shown for three biodiversity facets, respectively. In (J) to (L), the grid cells with more than 50% of total beta-diversity contributed by nestedness are shown in red.

Globally, the total beta-diversity and its turnover and nestedness components exhibited strikingly different spatial patterns across all three biodiversity facets (Fig. 2). There was relatively low spatial variation in total beta-diversity, with low values mainly concentrated in the eastern United States, southwestern Europe, and eastern South America (Fig. 2, A to C). By contrast, turnover and nestedness components showed clear but opposing latitudinal patterns (Fig. 2, D to I, and fig. S1). The turnover component decreased toward the poles, whereas the nestedness component increased toward the poles. On average, the turnover component contributed more to total beta-diversity (Fig. 2, J to L). However, the nestedness component contributed more than half in high latitudinal areas in North America and Eurasia, as well as in smaller areas elsewhere, mostly in the vicinity of deserts. This pattern was especially pronounced for phylogenetic and functional beta-diversity (Fig. 2, J to L).

Across three biodiversity facets, spatial patterns of total beta-diversity and its components of turnover and nestedness are generally consistent (Fig. 2), as indicated by strong positive correlations between taxonomic, phylogenetic, and functional beta-diversity (fig. S2). On average, the total taxonomic beta-diversity was greater than the total phylogenetic beta-diversity, which, in turn, exceeded the total functional beta-diversity; the same pattern was observed for the turnover component (fig. S2, A to F). However, phylogenetic and functional nestedness were greater than taxonomic nestedness, resulting in a higher relative contribution of the nestedness component to total phylogenetic and functional beta-diversity as compared to taxonomic beta-diversity (fig. S2, J to L).

### Effects of past climate stability and current environmental conditions

**Fig. 3.**
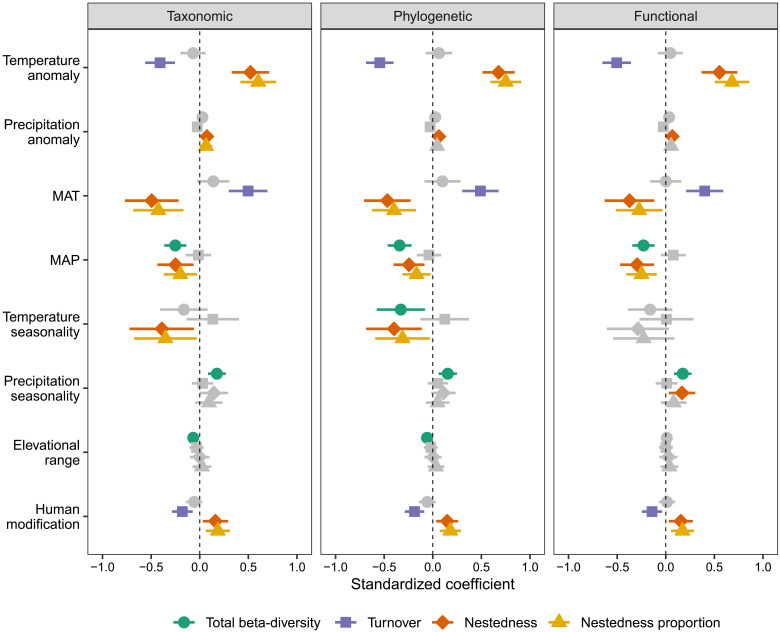
Effects of past climate stability and current environmental conditions on taxonomic, phylogenetic, and functional beta-diversity of angiosperm trees. Total beta-diversity and its components of turnover and nestedness and the nestedness proportion were indicated with different shapes and colors. The averaged estimates of standardized coefficients (points) and the 95% confidence intervals (bars) were obtained from spatial simultaneous autoregressive models. Nonsignificant variables are shown in gray. Temperate and precipitation anomaly: The differences in annual temperature and precipitation between the present and the LGM. MAT, mean annual temperature; MAP, mean annual precipitation.

The environmental variables affecting total beta-diversity differ from those affecting the turnover and nestedness components (Fig. 3, fig. S3, and table S1). Across all three biodiversity facets, total beta-diversity was positively associated with precipitation seasonality and negatively associated with mean annual precipitation (Fig. 3). Precipitation seasonality showed the strongest correlation with total beta-diversity (table S1). The turnover and nestedness components of all three biodiversity facets were most strongly influenced by temperature anomaly since the LGM and mean annual temperature (Fig. 3, fig. S3, and table S1). However, turnover and nestedness components presented contrasting associations (Fig. 3, fig. S3, and table S1). Consistent with our predictions, the turnover component was negatively associated with the LGM temperature anomaly and positively with mean annual temperature, whereas the nestedness component was positively associated with the LGM temperature anomaly and negatively with mean annual temperature (Fig. 3 and fig. S4). Both turnover and nestedness components of all three biodiversity facets had a stronger correlation with the LGM temperature anomaly than with mean annual temperature (table S1). In addition, human modification, as a measure of human pressures, had negative effects on the turnover component and positive effects on the nestedness component across all three biodiversity facets (Fig. 3). The nestedness component also had a weak positive relationship with precipitation anomaly since the LGM and a weak negative relationship with mean annual precipitation across all three biodiversity facets (Fig. 3). Temperature and precipitation seasonality also had weak effects on the nestedness component, but these effects were inconsistent across three biodiversity facets (Fig. 3). For the proportion of total beta-diversity contributed by the nestedness, the important associated factors were similar to those for the nestedness component (Fig. 3).

Across six continents, beta-diversity was primarily influenced by different environmental variables, which could be attributed to differences in environmental gradients among these continents (fig. S5). Despite this geographic variation, the effects of past climatic stability on both turnover and nestedness components of beta-diversity were largely consistent with our predictions (fig. S5). For the turnover component of beta-diversity, the LGM temperature anomaly had negative effects in Europe, North America, and Asia, and the LGM precipitation anomaly had negative effects in Australia and North America. For the nestedness component, the LGM temperature anomaly had positive effects in Europe, North America, Asia, and Australia, and the LGM precipitation anomaly had positive effects in Australia and North America. Although we did not detect significant associations between beta-diversity components and the LGM temperature or precipitation anomaly in two continents (South America and Africa), no associations were opposite to our predictions in any continents (fig. S5).

### Deviations of phylogenetic and functional beta-diversity and associated factors

**Fig. 4.**
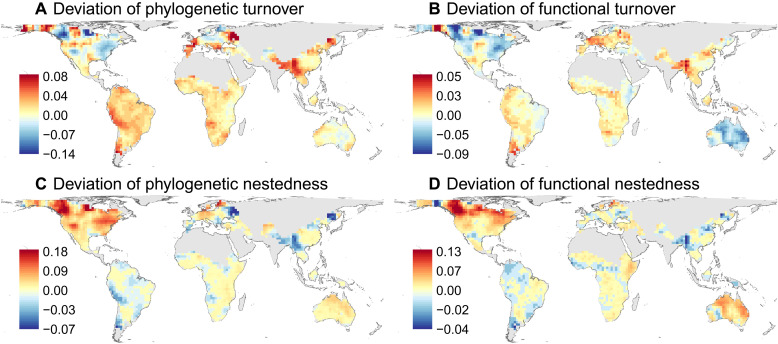
Global patterns of the deviation of phylogenetic and functional turnover and nestedness of angiosperm trees. The deviations of phylogenetic and functional turnover (**A** and **B**) and nestedness (**C** and **D**) were calculated as the differences between the observed and random expectations based on taxonomic beta-diversity and site-specific regional species pools.

Null model analyses showed that deviations of the observed phylogenetic and functional turnover and nestedness from random expectations based on observed taxonomic beta-diversity and site-specific regional species pools were not evenly distributed across space (Fig. 4). Positive deviations of phylogenetic and functional turnover were primarily concentrated in low-latitudinal and mountainous areas, particularly in southwest China and the Andes region, indicating that species replacement among assemblages largely occurred at deep phylogenetic branches and in species with dissimilar functional trait values. Meanwhile, most areas in North America had negative deviations in turnover, indicating the replacement of relatively recently evolved lineages and species with similar functional trait values. By contrast, positive deviations of phylogenetic and functional nestedness were mainly concentrated in high latitudinal areas, particularly in North America and some areas in Northern Europe. This pattern suggests phylogenetic and functional selection for closely related species and those with similar functional trait values in species losses (e.g., glaciation-driven extinction) or gains (e.g., postglacial recolonization). Nestedness deviations were usually close to zero for most other regions, suggesting phylogenetically and functionally random species losses or gains.

**Fig. 5.**
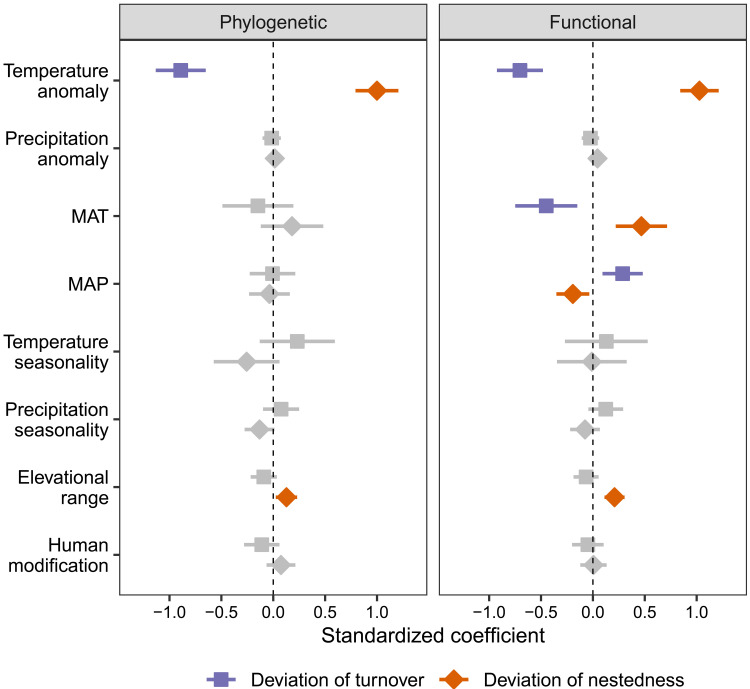
Effects of past climate stability and current environmental conditions on deviations of phylogenetic and functional turnover and nestedness of angiosperm trees. The deviations were calculated as the differences between the observed and random expectations based on taxonomic beta-diversity and site-specific regional species pools. The averaged estimates of standardized coefficients (points) and the 95% confidence intervals (bars) were obtained from spatial simultaneous autoregressive models. Nonsignificant variables are shown in gray. Temperate and precipitation anomaly: The differences in annual temperature and precipitation between the present and the LGM.

**Fig. 6.**
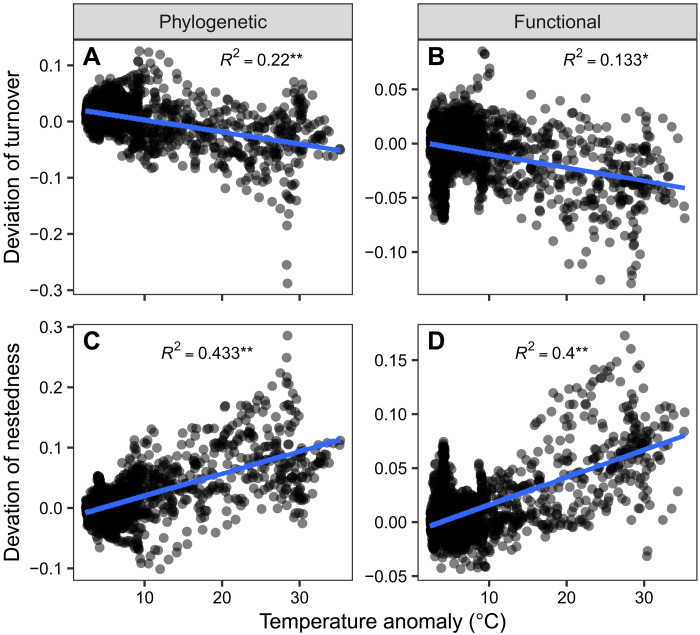
Relationships between temperature anomaly since the LGM and deviations of phylogenetic and functional turnover and nestedness of angiosperm trees. The deviations of phylogenetic and functional turnover (**A** and **B**) and nestedness (**C** and **D**) were calculated as the differences between the observed and random expectations based on taxonomic beta-diversity and site-specific regional species pools. The blue lines were fitted with linear regressions. Significance was tested using a modified *t* test to control for spatial autocorrelation. *R*^2^, coefficient of determination. *P < 0.05 and **P < 0.01.

Geographic variation in phylogenetic and functional turnover and nestedness deviation was most strongly associated with the LGM temperature anomaly (Fig. 5, fig. S6, and table S1). Consistent with our predictions, deviations of phylogenetic and functional turnover decreased with increasing LGM temperature anomaly, whereas deviations of phylogenetic and functional nestedness increased with increasing LGM temperature anomaly (Fig. 6). Specifically, there were negative turnover deviations and positive nestedness deviations in regions with large Quaternary temperature change. In contrast, no significant associations were detected between current climatic variables and deviations in phylogenetic turnover and nestedness. Deviations of functional turnover and nestedness were significantly associated with mean annual temperature and precipitation. Deviation of functional turnover was negatively associated with mean annual temperature and positively associated with mean annual precipitation, whereas deviation of functional nestedness was positively associated with mean annual temperature and negatively associated with mean annual precipitation (Fig. 5). Elevational range also had a weak positive relationship with both phylogenetic and functional nestedness (Fig. 5). Across continents, we found large variation in the effects of environmental variables on deviations of phylogenetic and functional turnover and nestedness (fig. S7). Consistent with global-scale analyses, the LGM temperature or precipitation anomaly was negatively associated with deviations of phylogenetic or functional turnover and positively associated with deviations of phylogenetic or functional nestedness in North America, Europe, and Asia (fig. S7).

### Sensitivity analyses

Patterns of functional beta-diversity shown in this study were robust to trait imputation despite a large proportion of species requiring missing traits to be imputed based on correlation structure among traits and phylogenetic information. Functional beta-diversity and its components of turnover and nestedness that were qualified based on all species had similar spatial patterns as those based on only species that have measured data for at least one or five traits (figs. S8 and S9), with Pearson’s correlations higher than 0.98 and 0.75, respectively (fig. S10). A reduced correlation between functional beta-diversity based on all species and those based on only species with at least five measured traits was likely the consequence of only a small subset of species within some grid cells being used in calculating beta-diversity (fig. S11). In addition, functional and phylogenetic beta-diversities were strongly correlated even when only species with at least one or five measured traits were used in calculating beta-diversity (fig. S12). Specifically, functional turnover was generally lower than phylogenetic turnover, and functional nestedness was similar to phylogenetic nestedness in magnitude; these patterns were consistent with the patterns based on all species (fig. S12). Furthermore, the functional and phylogenetic beta-diversity relationship based on all species was largely consistent across six continents (fig. S13). The correlation between functional and phylogenetic beta-diversity was not consistently weaker across beta-diversity metrics in Europe and North America, which had a higher trait data availability (figs. S11 and S13). This suggested that a strong correlation between functional and phylogenetic beta-diversity was not merely the consequence of trait imputation considering phylogenetic information.

Our results were also robust to the approach used to model tree ranges from distribution occurrences and to the size of moving windows used to calculate intraregional beta-diversity (figs. S14 to S21). Geographic patterns of taxonomic, phylogenetic, and functional beta-diversity shown in the main text were based on alpha-hull ranges estimated with an alpha value of 6 (see the “Tree distributions” section in Materials and Methods for details). These were very similar to those based on alpha-hull ranges estimated with different alpha values (i.e., 2, 4, and 10; figs. S14 to S16), as indicated by strong correlations (Pearson’s *r* > 0.95; fig. S17). Regarding the influence of sizes of moving windows, intraregional beta-diversity within moving windows of nine (3 × 3) grid cells (fig. S18) showed similar spatial patterns to those within moving windows of 25 (5 × 5) grid cells shown in the main text. There were also similar spatial patterns for phylogenetic and functional turnover deviations and nestedness (fig. S19). The effects of environmental variables on both observed beta-diversity and deviations of phylogenetic and functional turnover and nestedness based on moving windows of nine grid cells (figs. S20 and S21) were qualitatively consistent with those reported in the main text. Specifically, the LGM temperature anomaly was consistently associated with both turnover and nestedness components of all three biodiversity facets, i.e., a negative relationship with turnover and a positive relationship with nestedness (fig. S20). Although we did not detect a significant relationship between the LGM temperature anomaly and deviations of phylogenetic and functional turnover, a positive relationship remained between temperature anomaly and deviations of phylogenetic and functional nestedness (fig. S21).

## DISCUSSION

Our results demonstrate that Quaternary climate change plays a major role in shaping present-day patterns of the turnover and nestedness components of taxonomic, phylogenetic, and functional beta-diversity in angiosperm trees worldwide. Among the environmental variables examined here, temperature anomaly since the LGM had the strongest relationship with beta-diversity patterns, particularly for phylogenetic and functional biodiversity facets (see Figs. 3 and 5 and table S1). Consistent with our hypotheses (Fig. 1), the temperature anomaly showed a negative relationship with the turnover and a positive relationship with the nestedness components of beta-diversity, reflecting different ecological and evolutionary processes driven by Quaternary climate change. The effects of temperature anomaly were also clear at the continental scale, particularly in the Northern Hemisphere, which experienced a large temperature decrease during the LGM. These results are generally consistent with previous regional and global studies that mostly focused only on turnover and nestedness components of taxonomic beta-diversity in other organisms (e.g., amphibians, freshwater fish, mammals, and birds) (*17*–*19*, *29*), expanding the idea of the fundamental role played by past climate change in shaping biodiversity patterns to angiosperm trees worldwide. By extending this recognized relation to angiosperm trees, one of the ecologically most important organismal groups worldwide, our results highlight that strong climate change has the potential to notably influence global biodiversity and ecosystem properties not only via direct effects but also via its effects on this ecosystem-defining group. Although few large-scale studies have analyzed taxonomic beta-diversity together with phylogenetic and/or functional beta-diversity, they usually directly compared the observed values of different facets of beta-diversity (*14*, *15*, *37*–*39*). Our study differs from previous analyses by comparing phylogenetic and functional turnover and nestedness with those randomly expected given taxonomic beta-diversity. Using this novel approach, we found lower phylogenetic and functional turnover and higher nestedness than random expectations in regions that experienced large Quaternary temperature changes (Figs. 4 to 6), patterns that have not been reported before. This finding suggests that processes such as species replacement, extinction, and colonization during glacial-interglacial oscillations are phylogenetically and functionally selective. For instance, species from specific lineages or with specific traits are more likely to experience glaciation-driven extinction or lags on their postglacial recolonization.

There is a high nestedness component of beta-diversity in angiosperm trees in regions with large Quaternary climate change. Previous studies at smaller scales or for other organisms often explained this by strong local species extinction during glaciations and incomplete postglacial recolonization from ice-age refugia (*17*, *19*, *29*). These factors also provide a likely explanation for angiosperm trees. For instance, several studies have shown that geographic accessibility from glacial refugia accounts for much of the variation in tree diversity across Europe (*55*, *56*). In addition, time since deglaciation is strongly positively associated with regional species richness of vascular plants in the Arctic (*57*). These patterns are consistent with the evidence that the current distributions of many tree species in Europe and North America are not in equilibrium with the current climate, often due to postglacial dispersal limitations (*26*, *58*). Plant species with lower dispersal capacity tend to occupy lower proportions of their potential ranges, and their current distributions have stronger associations with accessibility to glacial refugia (*27*, *59*). Differential and limited dispersal capacity would result in steep rates of species loss from glacial refugia toward higher latitudes, leading to strong nestedness of assemblagecompositions in regions strongly affected by Quaternary climate change (*60*).

Because species differ in functional traits and evolutionary history, they may have differential capacities to survive during glaciations and to recolonize postglacial suitable areas (*27*, *50*, *59*, *61*). Specifically, cold tolerance and dispersal capacity are regarded as important species attributes affecting species survival during glaciations and recolonization to suitable postglacial areas, respectively (*27*, *50*, *59*, *61*). These attributes have also been shown to be phylogenetically conserved (*48*, *62*, *63*). Therefore, species from specific lineages with strong cold tolerance are more likely to survive during glaciations, and those from lineages with strong dispersal capacity are more likely to recolonize their postglacial climatically suitable areas (*27*, *48*). Because many traits are correlated with each other because of biophysical constraints and trade-offs among traits (*64*, *65*), species with strong cold tolerance and dispersal capacity are probably also affiliated with specific trait values for some other functional traits such as those used in this study (e.g., maximum height and seed dry mass), which would be selected during glacial-interglacial oscillations. The contemporary functional composition of plant assemblages measured with five traits (specific leaf area, plant height, seed mass, stem specific density, and leaf nitrogen concentration) is still influenced by the Quaternary climate in North and South America (*66*). This functionally selective process has also been shown in patterns of functional diversity of plants in Europe, with regions that experienced large Quaternary climate change usually associated with low fractions of maximum potential functional diversity based on current environmental conditions (*49*). Together, the phylogenetically and functionally selective processes will cause a lower phylogenetic and functional diversity than randomly expected from species richness for species-poor assemblages and drive a larger difference in phylogenetic and functional diversity (i.e., stronger nestedness structure) between species-rich and species-poor assemblages.

In contrast with the nestedness component, the turnover component of beta-diversity in angiosperm trees is high in regions with large Quaternary climate stability, reflecting the prevalence of species with small ranges (*22*–*25*). Long-term stable climatic conditions can benefit species persistence in situ and could increase speciation rates due to increased opportunities for regional niche differentiation (*21*, *32*). The accumulated species are expected to be generally specialized and have small ranges (*21*, *32*), leading to a high turnover of species across sites. By comparison, small-ranged species in regions with large Quaternary climate change experience disproportionate extinctions because they usually have low vagility and poor adaptation to changed conditions (*21*). Therefore, regions with large Quaternary climate change have a high proportion of large-ranged species (*22*–*25*), decreasing species replacement among neighboring sites. Our global analyses of angiosperm trees support the above theoretical expectations and are generally consistent with previous studies of other organisms or smaller scales (*17*–*19*, *29*), suggesting a prevalent paleoclimatic legacy on spatial turnover in biodiversity.

This worldwide study using angiosperm trees shows lower phylogenetic and functional turnover than randomly expected from taxonomic beta-diversity in regions with large Quaternary climate change. This suggests that species replacements between sites in regions with large Quaternary climate change usually stem from closely related young lineages with similar functional trait values (Fig. 1C). This finding is consistent with evidence that floras in environmentally unstable regions usually have more recent evolutionary divergence times (*44*). These floras also tend to be phylogenetically clustered, meaning that species are closely related to each other (*44*, *67*). Similarly, assemblages in regions with large Quaternary climate change usually have low functional dispersion, reflecting species compositions with similar functional trait values (*49*). However, the associations of Quaternary temperature change with deviations of phylogenetic and functional turnover were weaker than with deviations of phylogenetic and functional nestedness (Fig. 6), and these associations became insignificant in sensitivity analyses using the moving window size of nine grid cells (fig. S21). These differences suggest that the phylogenetically and functionally selective processes leading to higher phylogenetic and functional nestedness than random expectations (see above) are the main glaciation-driven processes that result in a departure of phylogenetic and functional beta-diversity from the taxonomic beta-diversity.

In addition to paleoclimate change, contemporary environmental conditions also play important roles in explaining global patterns of beta-diversity in angiosperm trees. Consistent with the major role of temperature in shaping plant beta-diversity across the Americas (*13*), annual temperature is the strongest current climatic variable to explain the turnover component of beta-diversity, with high turnover in regions with warm conditions. By comparison, the nestedness component is high in regions with cold conditions, likely reflecting strong filtering of species under stressful conditions, because most lineages probably originate in tropical climates and are difficult to adapt in freezing conditions [“tropical niche conservatism” hypothesis (*68*)]. The nestedness component also tends to be higher when annual precipitation is lower, likely because water availability is critical for trees, and species would be gradually filtered out in areas with limited water availability. In addition, while controlling for other variables, human pressures significantly impact tree beta-diversity patterns, decreasing the turnover component but increasing the nestedness component. This decreased turnover reflects the pattern of biotic homogenization driven by human activities as a consequence of the replacement of narrow-ranged species by widespread species (*36*, *69*). The increased nestedness probably suggests greater species losses in some sites than others, which reflect landscapes being differentially influenced by human activities such as deforestation (*70*).

Although generally similar, there were clear differences in spatial patterns of phylogenetic and functional beta-diversity and associations with environmental variables. First, functional turnover was generally lower than phylogenetic turnover, particularly in regions with high phylogenetic turnover (usually tropical regions; Fig. 2, E and F, and fig. S2F), suggesting that tree species among neighboring sites are more similar in functional trait values than in phylogenetic relationship. This pattern reflects functional redundancy among regional co-occurring species, which may result from environmental filtering selecting functionally similar species (*71*) and/or evolutionary convergence leading to similar traits among independent lineages (*72*). Second, we found significant effects of mean annual temperature and precipitation on deviations of functional turnover and nestedness but no effects of current climatic conditions on deviations of phylogenetic turnover and nestedness (Fig. 5). These differences suggest a stronger functional beta-diversity response to contemporary environmental conditions compared to phylogenetic beta-diversity. Although we observed differences in the phylogenetic and functional beta-diversity patterns, we note that these two measures are not independent in this study, because phylogenetic information was used in the imputation of missing functional traits. Increasing the amount of measured trait data is expected to result in greater differences between these two diversity dimensions. Because trait data coverage was not evenly distributed across regions, with higher coverage in Europe and North America (fig. 11), the functional beta-diversity could be more redundant with phylogenetic beta-diversity due to trait imputation in regions with poor trait coverage, leading to spatial uncertainties in spatial patterns of functional beta-diversity. This concern, however, is unlikely to be a major issue, because our sensitivity analyses using only species with at least one and five measured traits showed similar spatial patterns of functional beta-diversity as those using all species (figs. S8 to S10) and because a strong correlation between functional and phylogenetic beta-diversity was prevalent across continents regardless of trait coverage (fig. S13).

In summary, our results show that Quaternary climate change has left a strong legacy in current global patterns of taxonomic, phylogenetic, and functional beta-diversity for angiosperm trees, a keystone organismal group in terrestrial ecosystems. Notably, high levels of Quaternary temperature instability are associated with marked gradients in species, lineage, and trait diversity across sites, reflecting severe past losses, incomplete recolonization, and low levels of replacement in all three diversity facets. Furthermore, strong Quaternary climate change is also associated with lower phylogenetic and functional turnover and higher nestedness than randomly expected from taxonomic beta-diversity, reflecting phylogenetically and functionally selective processes during glacial-interglacial oscillations. These findings suggest that large Quaternary climate changes resulted in the reduction and homogenization of the diversity of angiosperm tree across large landscapes worldwide, with particularly severe effects on phylogenetic and functional composition. These diversity reductions are likely to affect ecosystem functions, such as plant productivity in forests and woodlands, because of the critical ecological importance of tree diversity (*51*, *73*). Our results highlight that future human-driven climate change is likely to have strong and long-lasting effects on tree assemblages globally via strong reduction and homogenization of taxonomic, phylogenetic, and functional diversity, potentially leading to impaired forest ecosystem functioning.

## MATERIALS AND METHODS

### Tree distributions

In this study, we used the global tree species list and distributions compiled in the TREECHANGE database (*53*). The tree species checklist came from the GlobalTreeSearch v.1.2 (*74*), which was assembled from a range of botanical publications and extended by many botanical experts. Taxonomic names were standardized using the Taxonomic Name Resolution Service (*75*), resulting in 54,020 tree species.

Tree species occurrences were compiled from five major comprehensive biodiversity infrastructures, including the Global Biodiversity Information Facility (GBIF; www.gbif.org) (*76*), the Botanical Information and Ecological Network v.3 (BIEN; http://bien.nceas.ucsb.edu/bien) (*77*), the Latin American Seasonally Dry Tropical Forest Floristic Network (DRYFLOR; www.dryflor.info) (*78*), the RAINBIO database (https://gdauby.github.io/rainbio/index.html) (*79*), and the Atlas of Living Australia (www.ala.org.au). These records were assessed using a quality control workflow considering geographic coordinates, duplications, native ranges, and geographical and environmental outliers and labeled from AAA (high geographic precision and low environmental uncertainty) to E (missing coordinates) (*53*). In this study, we used high-quality occurrences and those records without geographic bias distributed in native ranges, which were labeled as AAA, AA, A, and C [see (*53*) for further information]. The final dataset had 46,752 species with 7,066,785 occurrences at a resolution of 30 arc sec (fig. S22).

We then estimated species ranges using alpha hulls for species with 20 or more occurrences using the R package alphahull (*80*). The alpha hull is a generalization of the convex hull and allows the constructed geometric shape to consist of several discrete hulls dependent on the value of the parameter alpha (*81*). For species with fewer than 20 occurrences or disjunct records, a 10-km buffer was built around each point and then combined with alpha hulls. Following recommendations in previous studies (*82*), we used the alpha value of 6° to construct alpha hulls. To test the sensitivity of our results to alpha values, we also constructed alpha hulls using other alpha values and repeated analyses based on different range estimates (see the “Sensitivity analyses” section).

Species ranges were then rasterized to grid cells in a resolution of 200 km with an equal-area Behrmann projection using the R package letsR (*83*). Species assemblages in each grid cell were defined as all species with ranges falling within the grid cell. We chose the resolution of 200 km because biodiversity assessment at a coarse resolution can reduce the undersampling of species distributions. Although biodiversity patterns based on coarse species distributions probably underestimate the variation in species compositions at small spatial scales, this study focuses on global variation in beta-diversity at broad spatial scales and how past and contemporary macroclimate gradients determine these changes. We excluded gymnosperms from our analyses because including them can strongly influence plant phylogenetic structure due to deep split time between gymnosperms and angiosperms, which means whether there is a gymnosperm in a grid cell can strongly influence its phylogenetic diversity, obscuring patterns within angiosperms (*84*). We did not perform analyses for gymnosperms separately because there were only 579 gymnosperms in our dataset and 792 grid cells worldwide that had at least five gymnosperms (fig. S23). We removed grid cells with fewer than five species to avoid potentially unreliable beta-diversity estimates (see fig. S23 for spatial pattern of species richness). A total of 2319 assemblages and 43,635 species were kept and used in subsequent analyses.

### External validation of tree distributions

We performed three types of external validations to validate the alpha-hull ranges constructed in this study. The details of these external validations have been published in (*85*), which used the same range maps as those used in this study. First, we compared global patterns of species richness based on our range maps with predicted richness from an independent study that integrated 1336 forest plots and 282 regional checklists (*86*). Our estimated richness shows a similar spatial pattern to those predicted based on plots and regional checklists, although the predicted richness was generally higher than our estimates. Second, we compared our range maps with ranges from two external datasets: EU-Forest (*87*) and Little’s “Atlas of the United States trees” (*88*). EU-Forest includes range maps of 242 tree species in Europe based on 1,000,525 occurrence records, and Little’s Atlas includes ranges of 680 tree species in North America based on botanical lists, forest surveys, field notes, and herbarium specimens. There were 120 and 536 species in our dataset matched to EU-Forest and Little’s Atlas. We calculated a co-occurrence index to measure the extent to which range maps from our dataset and external datasets overlapped. The co-occurrence index varies from 0 (no overlap) to 1 (complete overlap). For the matched tree species between our dataset and EU-Forest and Little’s Atlas, 73 and 85% of species had co-occurrence values higher than 0.8, and the mean co-occurrence values were 0.84 and 0.92, with the SD as 0.16 and 0.11, respectively. This suggests that the range maps are well matched between our dataset and two external datasets. Third, we compared the species richness of 459 geographic regions worldwide based on our range maps with those based on subnational species lists (*89*). The two richness estimates were highly correlated (Pearson’s *r* = 0.89), and the map of residuals from their relationship showed that the two richness estimates were similar in most regions.

### Phylogenetic tree

The phylogenetic information for the tree species was extracted from the largest seed plant phylogeny currently available [the “ALLBM” tree in (*90*)]. This synthesis phylogeny was constructed by combining sequence data from GenBank with a backbone tree reflecting deep relationships (*91*) and adding species not found in GenBank based on their placement in the Open Tree of Life, which reflects previous knowledge about phylogenetic relationships and taxonomy (*90*). Because there were 5791 tree species (10.7% of a total of 54,020 species) missing in the phylogeny due to different taxonomic backbone lists, these missing species were manually added into the phylogeny based on the most recent common ancestor using “add.species.to.genus” function in the R package phytools (*92*), following the same approach used in (*90*) to add missing species. We then reduced this phylogeny by removing any species absent in our list of tree species with distribution data (46,752 species). The generated phylogeny was further pruned to contain only the angiosperm species used in this study. Although the generated phylogeny contains some polytomies, this is unlikely to bias the global analyses of phylogenetic beta-diversity. Generating a phylogeny for a group of species by pruning from a synthesis tree, such as “ALLBM” used in this study, has been widely used in ecological analyses, and it has been shown that the common community phylogenetic analyses based on a synthesis tree and a purpose-built resolved tree can produce consistent results (*93*).

### Functional traits

We used the trait product for trees from (*85*). Specifically, we used eight functional traits related to plant growth, survival and reproduction (*64*), including specific leaf area, leaf area, leaf dry matter content, leaf nitrogen concentration, leaf phosphorus concentration, wood density, seed dry mass, and plant maximum height. These traits are often used in functional diversity analyses of plants and have relatively good data coverage (*49*, *64*, *66*, *94*). These traits were selected from a total of 20 traits that were compiled for the TREECHANGE project (*85*) (see table S2 for all trait names). Trait observations were compiled from three databases, including TRY Plant Trait Database (www.try-db.org) (*94*), BIEN (*77*), and TOPIC (*95*). However, only 11,659 species (21.6% of 54,020 tree species) had data for at least one functional trait. The trait gaps were filled by Bayesian hierarchical probabilistic matrix factorization (BHPMF), which is a robust machine learning approach imputing trait values based on the taxonomic hierarchy and correlation structure among traits (*96*). Following the suggestion of the BHPMF approach, trait values were log-transformed before imputation, and imputed trait values were back-transformed. All of the 20 functional traits were used in the imputation to maximize benefits from the correlation structure among traits. To improve the estimation of missing trait values, phylogenetic information in the form of phylogenetic eigenvectors was also used as predictors (*97*). These phylogenetic eigenvectors were extracted from a principal coordinate analysis on the genus-level phylogenetic distance matrix (*98*). To determine the number of phylogenetic eigenvectors included in the trait imputation, an increasing number of phylogenetic eigenvectors were added as predictors. The predicted trait values were then evaluated using the root mean squared error (RMSE) (*96*). When the first six phylogenetic eigenvectors were included in the imputation, the RMSE was minimized to 0.087, which means a high overall prediction accuracy [see (*85*) for more details]. The maximum and minimum observed trait values were used as thresholds to constrain imputed data to avoid outliers in the imputation of missing values. For plant maximum height, a height of 2 m was used to replace imputed values lower than that, considering the definition of trees in the GlobalTreeSearch database (*74*).

We noted that although a large proportion of tree species is missing in measured trait values, 55.8% of 4157 genera and 88.9% of 253 families have data for at least one functional trait (table S2). In addition, a lack of trait data for a large proportion of species did not mean low coverage of trait data across regions. We thus calculated the proportion of species in each grid cell that had measured data for at least one, five, or all eight traits. Although all regions, except Europe, had a low proportion of species with all eight traits, a considerable proportion of species had at least one or five traits in most regions, particularly Europe and North America (fig. S11). For 78% of the grid cells (1818 of a total of 2319 grid cells), more than half (>50%) of their species had at least one measured trait data. Therefore, our functional trait dataset provides substantial information about trait variation across species in most regions.

We further tested phylogenetic signals of eight traits for species with measured trait data. We calculated phylogenetic signals as Pagel’s λ using the “phylosig” function in the R package phytools (*92*). Pagel’s λ can range from zero to one, with zero indicating no phylogenetic signal and one indicating the strongest phylogenetic signal (*99*). We found that all eight traits had significant phylogenetic signals (*P* < 0.001), with lambda values ranging from 0.602 to 0.954 (table S3). This supported our approach using phylogenetic relatedness to predict traits. We also tested phylogenetic signals of traits using imputed trait data. Because all species had imputed traits and the number of species may influence the phylogenetic signal estimates, we calculated the number of species that had measured data for each trait and randomly sampled this number of species from the imputed traits. We repeated this random sampling 200 times and calculated the mean and SD of phylogenetic signals across iterations. All eight imputed traits had stronger phylogenetic signals than the respective measured traits (table S3). This result was not unexpected because phylogenetic information was used to impute missing functional traits. This suggests that the imputed traits contain some redundant information with the phylogenetic tree, and thus the results of phylogenetic and functional analyses would be dependent to some degree due to trait imputation. To assess the influence of trait imputation, we performed sensitivity analyses using only species that had measured trait data for calculating phylogenetic and functional beta-diversity and compared relationships between phylogenetic and functional beta-diversity across six continents (see the “Sensitivity analyses” section).

### Beta-diversity

We used a moving window, containing 25 (5 × 5) grid cells of 200 km by 200 km, to measure intraregional compositional heterogeneity as beta-diversity for each focal cell (*13*, *29*). As a sensitivity analysis, we further used a moving window of nine (3 × 3) grid cells to measure intraregional beta-diversity (see the “Sensitivity analyses” section). In this study, we used the multiple-site dissimilarity, rather than the averaged pairwise dissimilarity between a focal cell and its neighboring cells, because the averaged pairwise dissimilarity cannot reflect co-occurrence patterns among more than two sites (*100*). Because the number of grid cells within a region can affect the multiple-site dissimilarity (*16*) and some cells on islands and along the margins of continents have few neighboring cells, we only included grid cells with at least 13 neighboring cells as focal cells (leaving a total of 1838 grid cells) to calculate beta-diversity. For each focal cell, we further randomly sampled 13 cells from its 24 neighboring cells and used them together with the focal cell to calculate multiple-site dissimilarity. We performed this resampling 200 times and calculated the average beta-diversity.

We calculated taxonomic, phylogenetic, and functional beta-diversity using indices from the family of Sørensen-based multiple-site dissimilarity measures (*16*, *40*). As one of the most commonly used taxonomic dissimilarity indices, Sørensen dissimilarity index was used to measure the proportion of exclusive species among assemblages. The analogs of Sørensen dissimilarity index for phylogenetic and functional beta-diversity were used to measure the proportion of exclusive branch lengths in a phylogenetic tree or functional dendrogram for the species among assemblages (*101*). We then partitioned the three facets of Sørensen dissimilarity into two additive components due to spatial turnover and nestedness (*16*, *40*). The turnover component, which is the Simpson dissimilarity, reflects the effect of replacements of species or branches in a phylogeny or functional dendrogram without the impact from differences in species richness, phylogenetic, and functional diversity among sites (*16*, *40*). The nestedness component, which is the difference between Sørensen and Simpson dissimilarities, reflects the contribution due to differences in species richness, phylogenetic, and functional diversity when species-poor assemblages are nested in species-rich assemblages (*16*, *40*). All calculations of beta-diversity were performed using the R package betapart (*54*). We noted that although some other indices can be used to measure different patterns of dissimilarity between assemblages, these indices were usually related to Sørensen and Simpson dissimilarity (*102*).

Although functional beta-diversity can be calculated based on the volume of intersections between convex hulls in a multidimensional functional space (*41*), our calculation was based on a functional dendrogram because it has a similar data structure to the phylogenetic tree, making functional and phylogenetic beta-diversity comparable (*103*), and because the convex hull–based method is sensitive to extreme trait values or outliers and requires very long computation time, particularly for assemblages with many species similar to ours (*41*, *104*). We performed a principal components (PCs) analysis on all eight log- and z-transformed trait values to remove the correlation structure among traits. The first four PCs explained a high amount (93.5%) of the total variation. We thus used these four PCs to calculate the Euclidean distances among species and performed hierarchical clustering to generate a functional dendrogram based on the method of “complete” using the R package fastcluster (*105*).

To reflect the relative importance of turnover and nestedness components, we also calculated the proportion of total beta-diversity contributed by the nestedness component for all three evaluated biodiversity facets (*15*, *19*, *39*). Proportions lower than 0.5 indicate that total beta-diversity is mainly determined by turnover, whereas proportions higher than 0.5 suggest that the nestedness-resultant dissimilarity is the main component.

### Null model

Because the three facets of beta-diversity are not independent and usually strongly correlated, the processes driving taxonomic beta-diversity also affect phylogenetic and functional beta-diversity (*38*–*41*). To investigate whether phylogenetic and functional beta-diversity are affected by processes beyond those shaping taxonomic beta-diversity, we constructed a null model to calculate the random expectations of phylogenetic and functional beta-diversity based on the observed taxonomic beta-diversity and site-specific regional species pools. For each focal cell, we defined the site-specific regional species pool as all species in the 25-cell moving window that was used to calculate the beta-diversity for the given focal cell. In the null model, the identities of species were randomly shuffled among the species pool in the phylogenetic tree and functional dendrogram. Therefore, species richness of each grid cell, intraregional taxonomic beta-diversity and its components of turnover and nestedness, as well as the pool of species branches in the phylogenetic tree and functional dendrogram were kept constant with the observed in the null model.

Because this study focused on whether there are some processes driving stronger or weaker intraregional turnover and nestedness patterns in phylogenetic and functional compositions than expected by chance, we calculated the random expectations of phylogenetic and functional turnover and nestedness. We repeated our null model 200 times and calculated the average random expectations. Then, we calculated the deviations of phylogenetic and functional turnover and nestedness as the differences between the observed and the average expected values.

### Environmental data

To measure past climate stability, we calculated the change in mean annual temperature and precipitation between the present and the LGM, defined as the temperature anomaly and precipitation anomaly. Contemporary environmental conditions were represented by current climatic conditions, topography, and human pressure. We note that topography may also capture the effects of past climate stability because topographic heterogeneity favors species to track climate change by shifting short altitudinal ranges (*23*). We included mean annual temperature and precipitation because of their roles in determining species distributions (*106*). Temperature and precipitation seasonality were also included because they were important drivers of species range size (*24*, *25*), which was directly related to beta-diversity. Temperature seasonality was measured as the SD of monthly temperature (bio4), and precipitation seasonality was measured as the coefficient of variation of monthly precipitation (bio15). Topography was measured as the elevational range within a grid cell using elevation data in a spatial resolution of 1 km. Human pressure was represented with the human modification index, which was modeled based on 13 anthropogenic stressors, considering different types of human activities (e.g., human settlement, agriculture, and energy production) (*107*). Human modification values range from 0 to 1, with higher values indicating stronger human pressures.

The temperature and precipitation data at the LGM were extracted from the PaleoClim database with a resolution of 5 arc min (*108*). The contemporary climate and elevation data were downloaded from the WorldClim v1.4 database, with a resolution of 5 arc min for climate (*109*). The human modification map was accessed from (*107*), with a resolution of 1 km. We first calculated the mean values of all climatic variables and human modification for each grid cell of 200 km. To match the scale of beta-diversity and environmental variables (*13*), the environmental conditions for each cell were further measured as the average of the focal cell and its 24 neighboring cells (fig. S24).

### Statistical analysis

We used the Pearson correlation to check pairwise relationships among environmental variables and also among multiple dimensional beta-diversity. To account for the spatial autocorrelation of these variables, a spatially corrected *t* test was used to assess statistical significance (*110*). Following suggestions in (*29*), we performed piecewise regressions to examine latitudinal patterns of beta-diversity and its turnover and nestedness components.

We then tested the effects of environmental variables on the global-scale variation in each of the beta-diversity variables (three facets of total beta-diversity and turnover and nestedness components, and deviations of phylogenetic and functional turnover and nestedness). First, we evaluated relationships between beta-diversity and eight environmental variables using bivariate linear regressions and the Pearson correlation. We log-transformed elevational range and square root–transformed human modification to improve these variables' normality and the regressions' linearity. Significances were assessed using a spatially corrected *t *test (*110*). Then, multiple ordinary least squares (OLS) linear regressions were used to calculate standardized regression coefficients and determine the relative importance of environmental variables using both the scaled beta-diversity and environmental variables. Residuals of OLS models, however, showed strong spatial autocorrelation, which could affect significance test and bias parameter estimates (*111*). To account for spatial autocorrelation, we used spatial simultaneous autoregressive (SAR) models that include a spatial weight matrix as an additional error term (*112*). We run SAR models with a range of neighbor distances from 200 to 1000 km in a step of 100 km and row-standardized coding style to define the spatial weight matrix and used the minimum residual spatial autocorrelation (minRSA) to select the most appropriate SAR model for each response variable (*112*). The criteria of minRSA is to minimize the summed absolute values of Moran’s *I* of the first 20 distance classes in the model residuals. For all beta-diversity variables, the SAR model with a neighbor distance of 300 km produced a minimal residual spatial autocorrelation and a lowest Akaike information criterion value. The final SAR models substantially reduced spatial autocorrelation in residuals relative to the OLS models (table S4). In the multiple regression models, we included all eight environmental variables to estimate their relative importance. Because of a strong correlation between mean annual temperature and temperature seasonality (fig. S25), we also fit models without temperature seasonality in a preliminary analysis. These models estimated a very similar coefficient for the mean annual temperature with that from models with all environmental variables, suggesting that our models were not sensitive to the strong correlation between environmental variables.

We also used Akaike-based model selection and multimodel inference to qualify the relative importance of each environmental variable by assessing all subsets of full SAR models (*113*). The Akaike weight of each model was first calculated. Then, we averaged the standardized regression coefficients for each variable across all evaluated models by weighting each value with the Akaike weight of the model that contained it. We also measured the importance of each environmental variable by summing the weights of all models including that variable.

To assess the variation in the effects of environmental variables on beta-diversity across continents, we further fit SAR models regressing each of beta-diversity variables against eight environmental variables within each of six continents (Africa, Asia, Australia, Europe, North America, and South America). We calculated the averaged standardized regression coefficients based on multimodel inference to estimate the relative effects of environmental variables. All analyses were performed in R using the packages spatialreg and spdep (*114*) for SAR models, MuMIn (*115*) for multimodel inference, and SpatialPack (*116*) for the spatially corrected *t *test.

### Sensitivity analyses

To test the influence of trait imputation on functional beta-diversity and its relationship with phylogenetic beta-diversity, we repeated the calculation of functional and phylogenetic beta-diversity using only species that have measured data for at least one or five traits. Because only 114 species had measured data for all eight traits, we did not perform a sensitivity analysis using only these species. We calculated correlations of functional and phylogenetic beta-diversity based on all species with those based on species with at least one or five measured traits. We also analyzed the relationship between functional and phylogenetic beta-diversity using values based on all species and only species with at least one and five measured traits, respectively, and assessed whether the relationship based on all species was consistent with that based on only species with some measured trait data. Because Europe and North America had higher trait coverage than other continents (fig. S11), the functional beta-diversity within these continents was less likely influenced by trait imputation. Thus, its relationship with phylogenetic beta-diversity tends to reflect their intrinsic association rather than the consequence of trait imputation. We, therefore, performed an analysis by comparing relationships between phylogenetic and functional beta-diversity within each of the six continents (Africa, Asia, Australia, Europe, North America, and South America) to test whether they were less strongly correlated in Europe and North America.

We then tested the sensitivity of our results to the values of the parameter alpha in constructing alpha-hull ranges. The alpha parameter determines the resolution of constructed alpha hulls, with a smaller value representing a higher resolution (*81*, *82*). When alpha approaches zero, the constructed shape is close to the original point set, whereas the constructed alpha hulls will be the typical convex hull when alpha takes infinite values. In the main text, we reported results based on alpha-hull ranges estimated with the alpha value of 6°. As a sensitivity analysis, we calculated beta-diversity based on alpha-hull ranges estimated with alpha values of 2°, 4°, and 10° and compared them with beta-diversity values reported in the main text.

To test the sensitivity of our results to the sizes of moving windows to define regions, we repeated analyses using a moving window of nine (3 × 3) grid cells. To control for different numbers of cells within regions, we only included grid cells with at least five neighboring cells as focal cells to calculate beta-diversity. For each focal cell, we resampled five grid cells from its eight neighboring cells 200 times and calculated the average values for all metrics of beta-diversity. We also calculated the deviation of phylogenetic and functional turnover and nestedness from their random expectations using the null model in our main analyses. Here, the site-specific regional species pool needed for the null model was all species in the nine-cell moving window for each focal cell. Effects of environmental variables on spatial variation in beta-diversity were then assessed using the same methods used in our main analyses.
