# Supplementary material for: Global beta-diversity of angiosperm trees is shaped by Quaternary climate change
Source: Sci Adv. 2023 Apr 5;9(14):eadd8553. doi: 10.1126/sciadv.add8553 (PMC10075971; doi:10.1126/sciadv.add8553)
Supplement: Supplementary file 1 — Figs. S1 to S25 Tables S1 to S4 [file sciadv.add8553_sm.pdf]

Science Advances
AAAS

Supplementary Materials for

Global beta-diversity of angiosperm trees is shaped by Quaternary climate change

Wu-Bing Xu *et al.*

Corresponding author: Wu-Bing Xu, wbingxu@gmail.com; Alejandro Ordonez, alejandro.ordonez@bio.au.dk

Sci. Adv. **9**, eadd8553 (2023)
DOI: 10.1126/sciadv.add8553

This PDF file includes:

Figs. S1 to S25
Tables S1 to S4

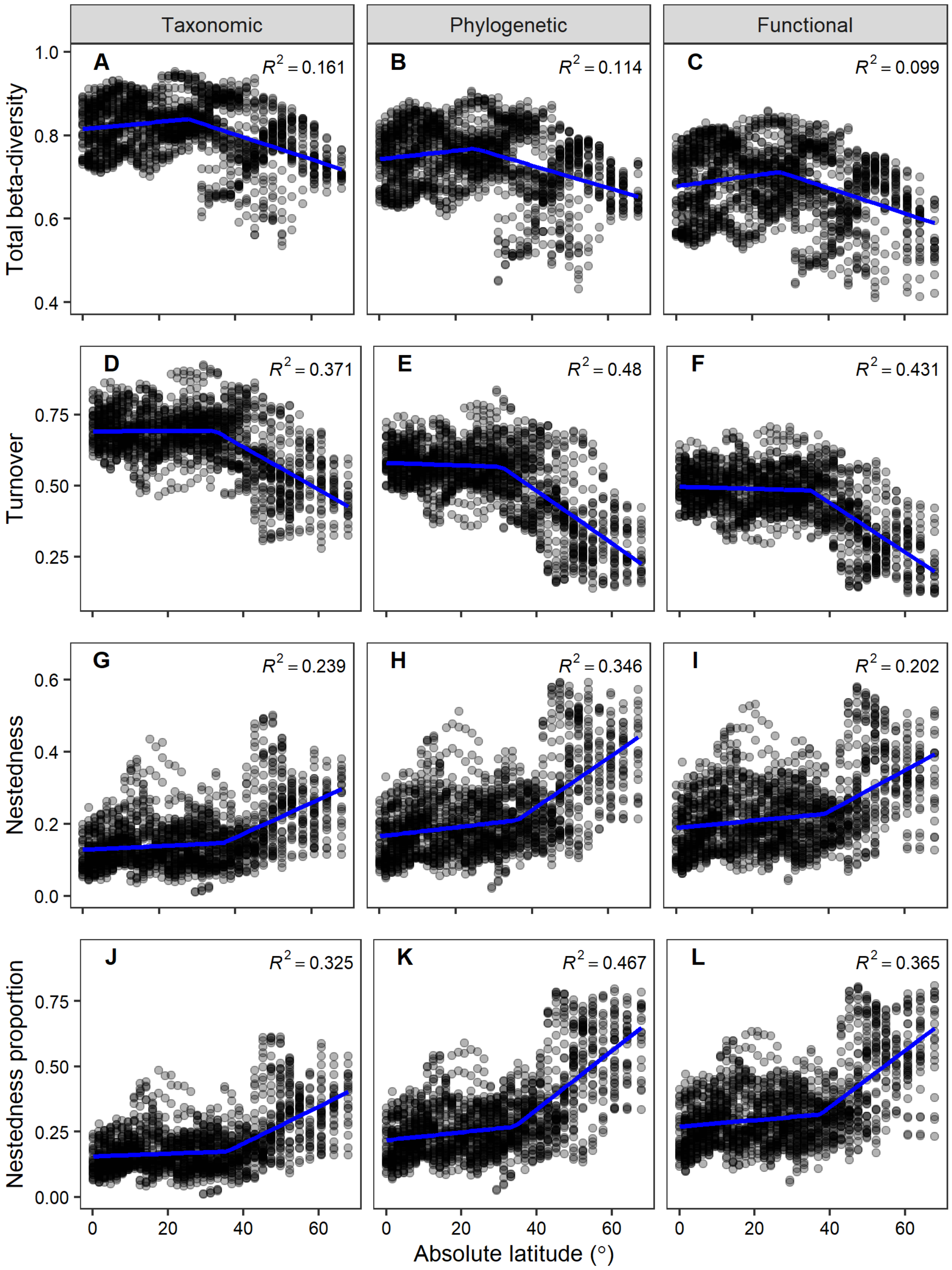

Fig. S1. Latitudinal patterns of taxonomic, phylogenetic and functional beta-diversity of angiosperm trees. Total beta-diversity (**A-C**) and its components of turnover (**D-F**) and nestedness (**G-I**) and the nestedness proportion (**J-L**) are shown for three biodiversity facets, respectively. The blue lines were fitted with piecewise regressions. R^2 was the squared Pearson correlation of predicted and observed values.

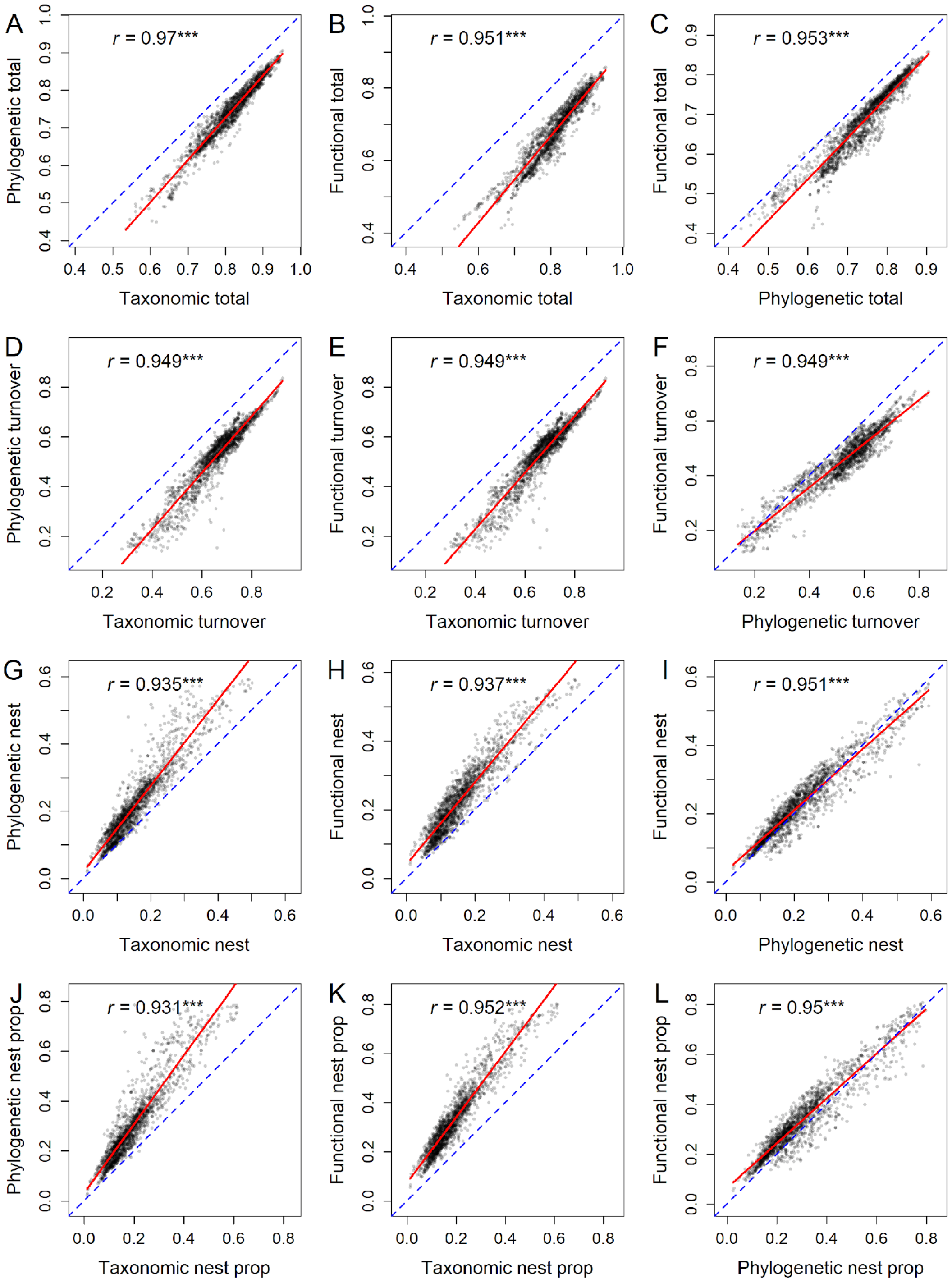

Fig. S2. Relationships among taxonomic, phylogenetic and functional beta-diversity of angiosperm trees. (**A-C**) Relationships for the total beta-diversity; (**D-F**) Relationships for the component attributable to spatial turnover; (**G-I**) Relationships for the component attributable to spatial nestedness; (**J-L**) Relationships for the proportion of total beta-diversity contributed by the nestedness component. The red lines were fitted with linear regressions. The blue dashed lines are the identity lines. Pearson's correlation coefficients were reported. Significance was tested using a modified *t*-test to control for spatial autocorrelation. ***, $P < 0.001$.

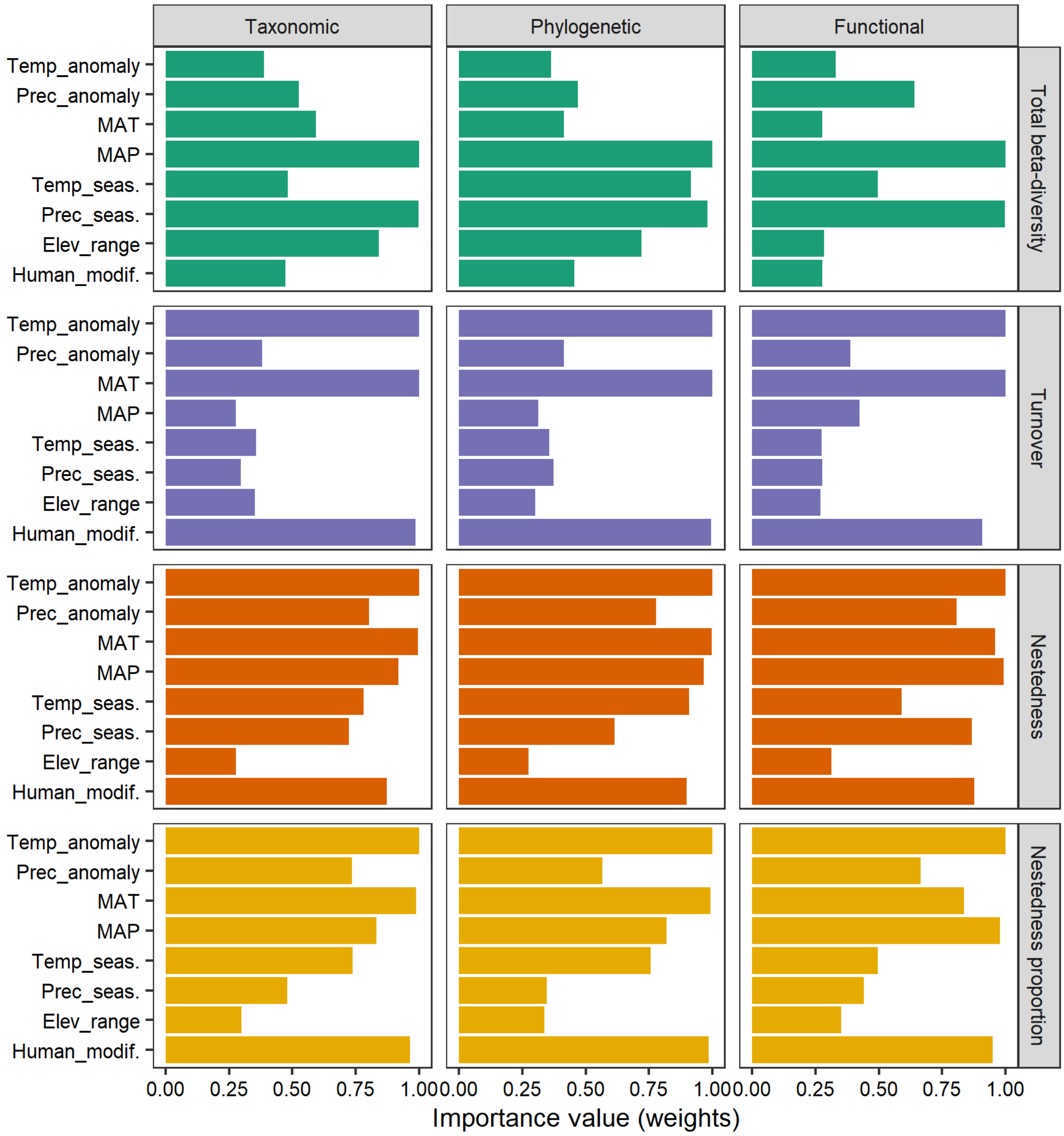

Fig. S3. The importance value of eight environmental variables in explaining spatial variations of taxonomic, phylogenetic and functional beta-diversity of Angiosperm trees. The importance value was measured as the summed Akaike weights of all subsets of full SAR models containing one specific variable. Total beta-diversity and its components of turnover and nestedness and the nestedness proportion are measured for three biodiversity facets, respectively. Temp_anomaly: temperature anomaly since the Last Glacial Maximum; Precp_anomaly: Precipitation anomaly since the Last Glacial Maximum; MAT: mean annual temperature; MAP: mean annual precipitation; Temp_seas.: temperature seasonality; Prec_seas.: precipitation seasonality; Elev_range: elevational range; Human_modif.: Human modification index.

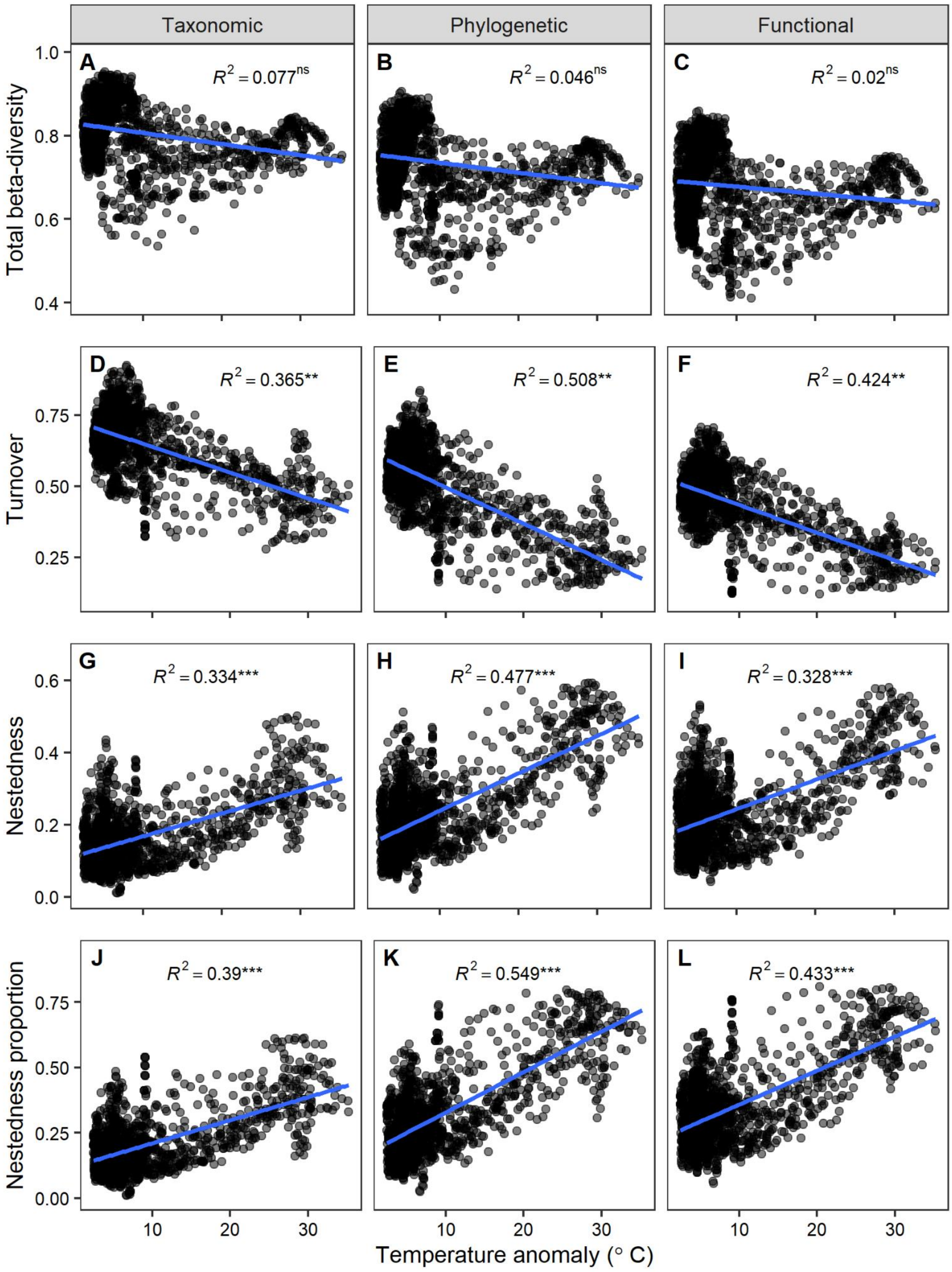

Fig. S4. Relationships between temperature anomaly since the Last Glacial Maximum and taxonomic, phylogenetic and functional beta-diversity of angiosperm trees. Total beta-diversity (**A-C**) and its components of turnover (**D-F**) and nestedness (**G-I**) and the nestedness proportion (**J-L**) are shown for three biodiversity facets, respectively. The blue lines were fitted with linear regressions. Significance was tested using a modified *t*-test to control for spatial autocorrelation.

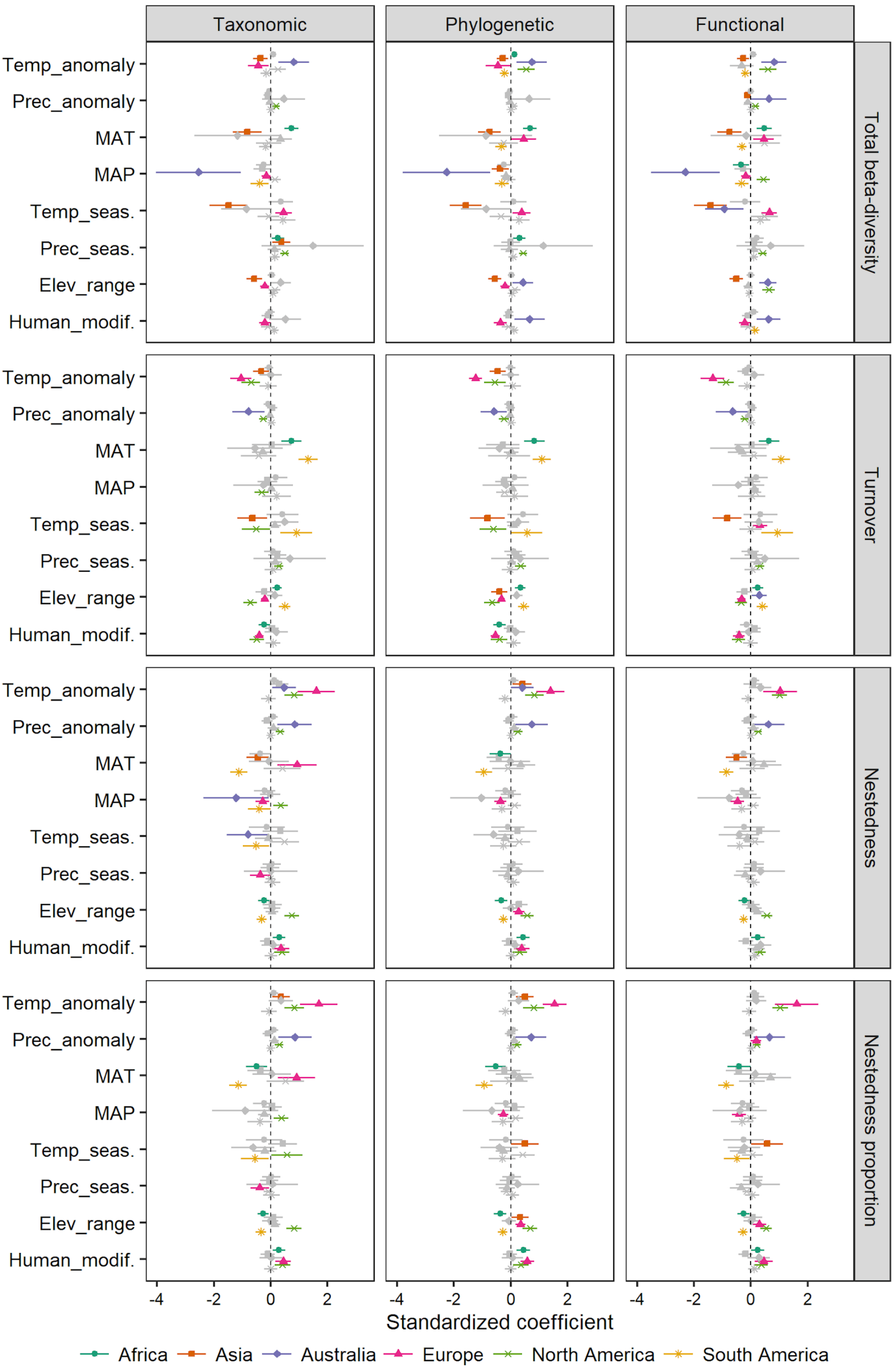

Taxonomic
Phylogenetic
Functional
Total beta-diversity
Turnover
Nestedness
Nestedness proportion
Temp_anomaly
Prec_anomaly
MAT
MAP
Temp_seas.
Prec_seas.
Elev_range
Human_modif.
-4
-2
0
2
Standardized coefficient
Africa
Asia
Australia
Europe
North America
South America

Fig. S5. Effects of past climate stability and current environmental conditions on taxonomic, phylogenetic and functional beta-diversity of angiosperm trees within six continents. Total beta-diversity and its components of turnover and nestedness and the nestedness proportion were analyzed, respectively. The averaged estimates of standardized coefficients (points) and the 95% confidence intervals (bars) were obtained from spatial simultaneous autoregressive models. Non-significant variables are shown in gray. Temp_anomaly: temperature anomaly since the Last Glacial Maximum; Precp_anomaly: Precipitation anomaly since the Last Glacial Maximum; MAT: mean annual temperature; MAP: mean annual precipitation; Temp_seas.: temperature seasonality; Prec_seas.: precipitation seasonality; Elev_range: elevational range; Human_modif.: Human modification index.

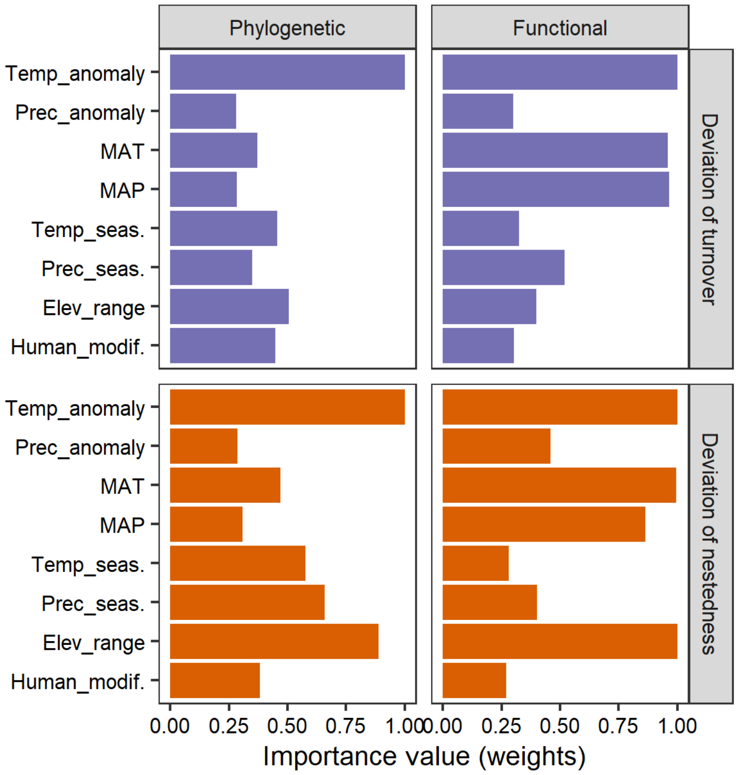

Fig. S6. The importance value of eight environmental variables in explaining spatial variations of the deviation of phylogenetic and functional turnover and nestedness. The deviations of phylogenetic and functional turnover and nestedness were calculated as the differences between the observed and random expectations based on taxonomic beta-diversity and site-specific regional species pools. The importance value was measured as the summed Akaike weights of all subsets of full SAR models containing one specific variable. Temp_anomaly: temperature anomaly since the Last Glacial Maximum; Precp_anomaly: Precipitation anomaly since the Last Glacial Maximum; MAT: mean annual temperature; MAP: mean annual precipitation; Temp_seas.: temperature seasonality; Prec_seas.: precipitation seasonality; Elev_range: elevational range; Human_modif.: Human modification index.

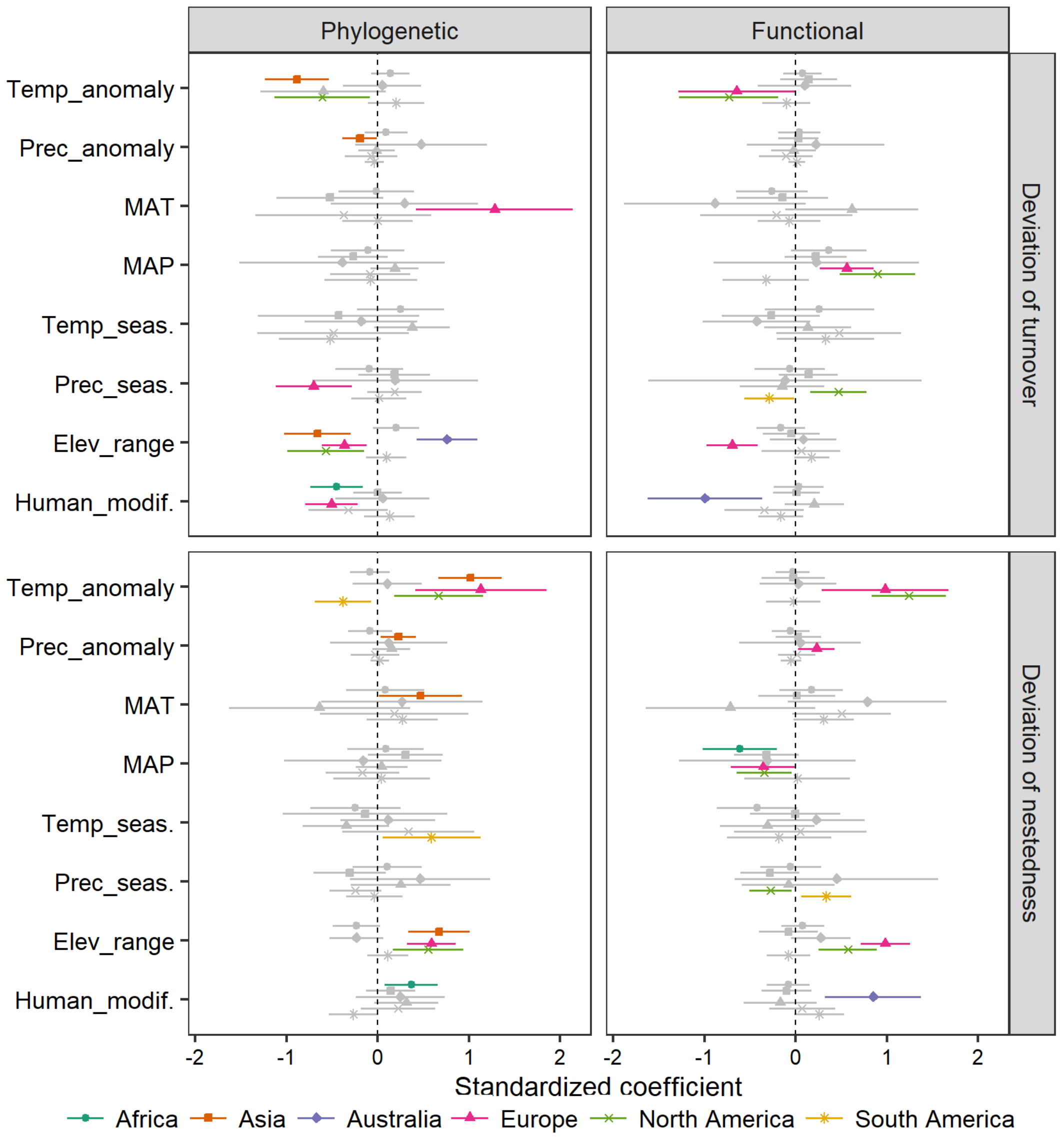

Fig. S7. Effects of past climate stability and current environmental conditions on deviations of phylogenetic and functional turnover and nestedness within six continents for angiosperm trees. The deviations were calculated as the differences between the observed and random expectations based on taxonomic beta-diversity and site-specific regional species pools. The averaged estimates of standardized coefficients (points) and the 95% confidence intervals (bars) were obtained from spatial simultaneous autoregressive models. Non-significant variables are shown in gray. Temp_anomaly: temperature anomaly since the Last Glacial Maximum; Precp_anomaly: Precipitation anomaly since the Last Glacial Maximum; MAT: mean annual temperature; MAP: mean annual precipitation; Temp_seas.: temperature seasonality; Prec_seas.: precipitation seasonality; Elev_range: elevational range; Human_modif.: Human modification index.

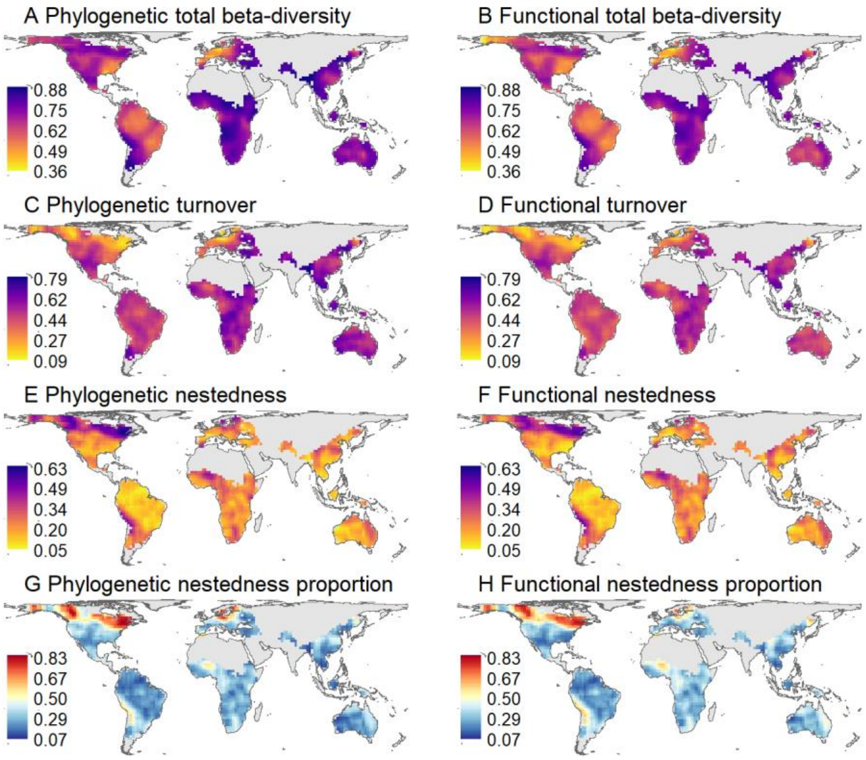

Fig. S8. Global patterns phylogenetic and functional beta-diversity of angiosperm trees calculated using species with at least one measured functional trait. Total beta-diversity (**A**-**B**) and its components of turnover (**C**-**D**) and nestedness (**E**-**F**) and the proportion of total beta-diversity contributed by nestedness (**G**-**H**) are shown for phylogenetic and functional facets, respectively. In **G**-**H**, the grid-cells with more than 50% of total beta-diversity contributed by nestedness are shown in red.

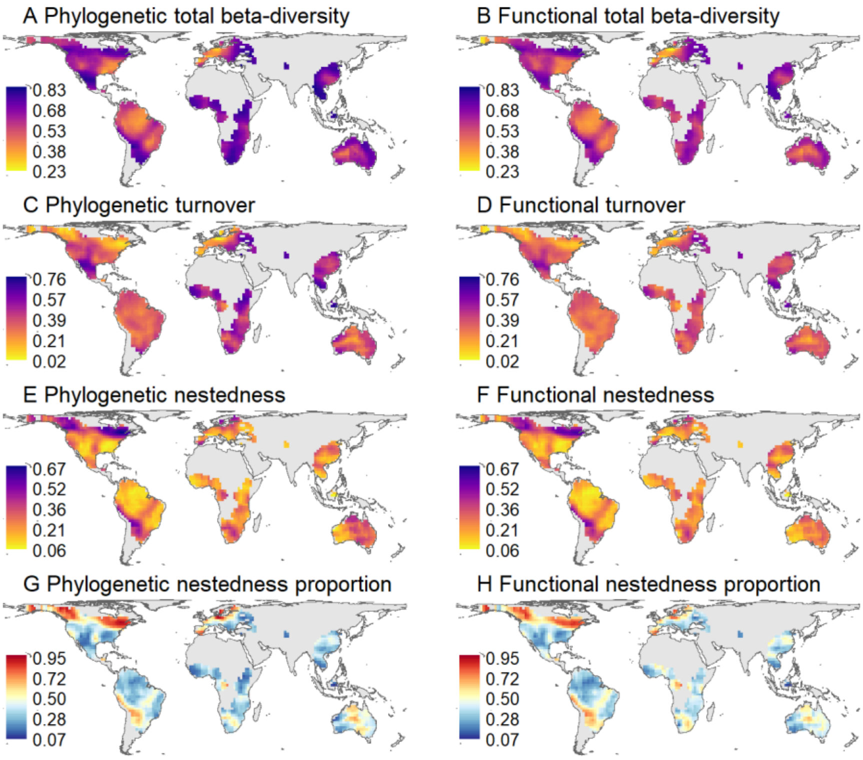

Fig. S9. Global patterns phylogenetic and functional beta-diversity of angiosperm trees calculated using species with at least five measured functional traits. Total beta-diversity (**A**-**B**) and its components of turnover (**C**-**D**) and nestedness (**E**-**F**) and the proportion of total beta-diversity contributed by nestedness (**G**-**H**) are shown for phylogenetic and functional facets, respectively. In **G**-**H**, the grid-cells with more than 50% of total beta-diversity contributed by nestedness are shown in red.

	A All species vs >= 1 traits		B All species vs >= 5 traits	
	Phylogenetic	Functional	Phylogenetic	Functional
Total	0.95	0.99	0.82	0.86
Turnover	0.95	0.98	0.68	0.75
Nestedness	0.98	0.98	0.78	0.83
Nestedness proportion	0.97	0.98	0.73	0.78

Fig. S10. Pearson's correlation coefficients between beta-diversity calculated using all angiosperm tree species and those with measured functional traits. Species with at least one or five measured functional traits were used in calculating beta-diversity in (**A**) and (**B**), respectively. The comparisons were performed for total phylogenetic and functional beta-diversity and their respective components of turnover and nestedness and the proportion of total beta-diversity contributed by nestedness, respectively.

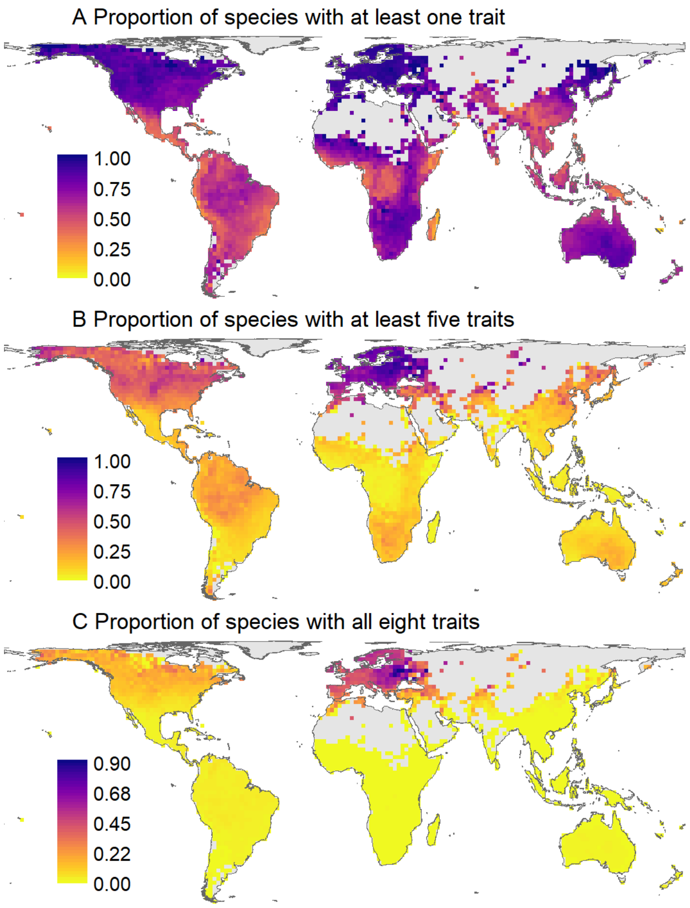

Fig. S11. Global patterns of trait coverage of angiosperm trees. The trait coverage was measured as the proportion of angiosperm tree species in each grid cell that have measured data for at least one (**A**), five (**B**) or all eight (**C**) functional traits which were used in this study. The grid cells with no data or fewer than five species are shown in gray.

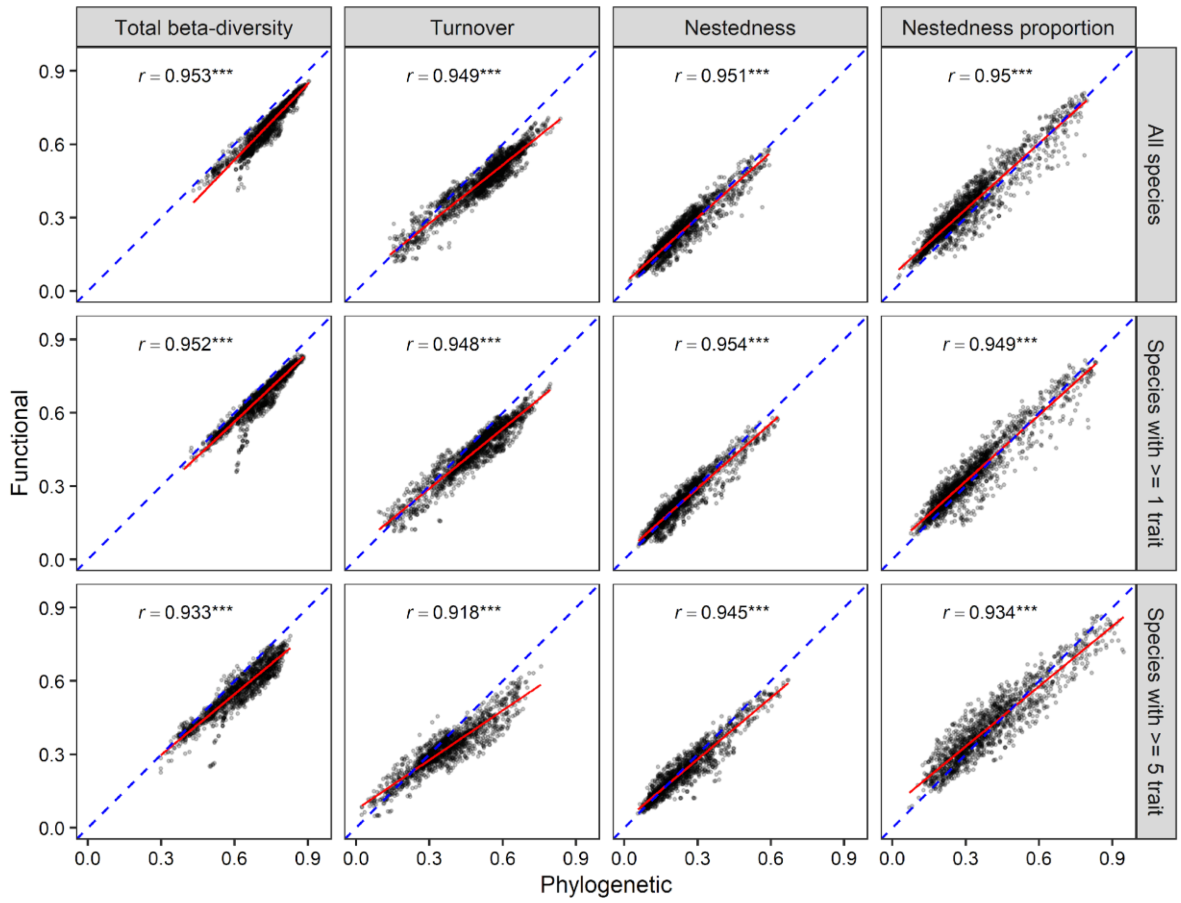

Fig. S12. Relationships between phylogenetic and functional beta-diversity calculated using all angiosperm tree species or those with at least one or five measured functional traits. The relationship was analyzed for total beta-diversity and the components of turnover and nestedness and the proportion of total beta-diversity contributed by nestedness, respectively. The red lines were fitted with linear regressions. The blue dashed lines are the identity lines. Pearson's correlation coefficients were reported. Significance was tested using a modified *t*-test to control for spatial autocorrelation. ***, $P < 0.001$.

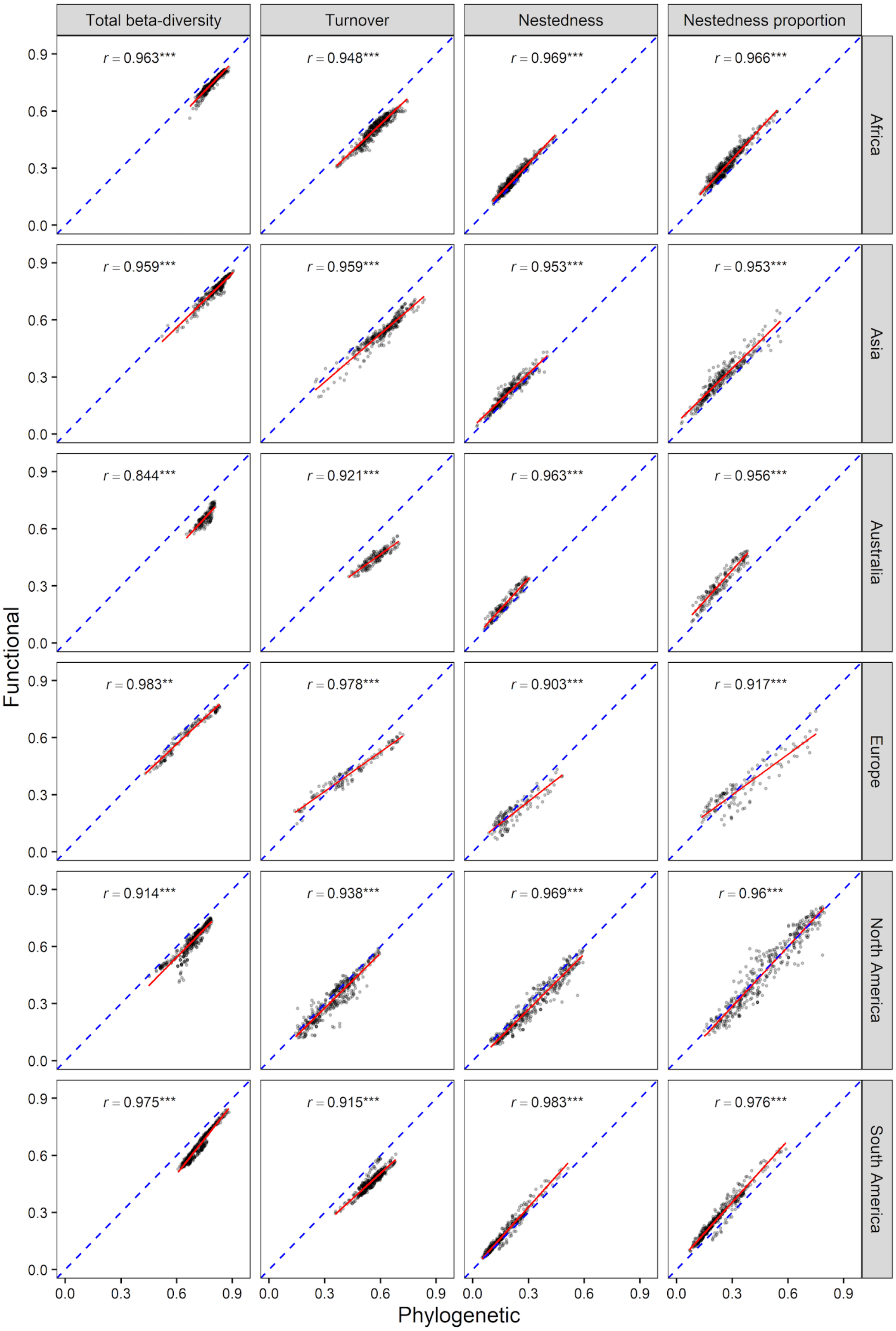

Fig. S13. Relationships between phylogenetic and functional beta-diversity within six continents. The relationship was analyzed for total beta-diversity and the components of turnover and nestedness and the proportion of total beta-diversity contributed by nestedness, respectively. The red lines were fitted with linear regressions. The blue dashed lines are the identity lines. Pearson's correlation coefficients were reported. Significance was tested using a modified *t*-test to control for spatial autocorrelation. ***, $P < 0.001$.

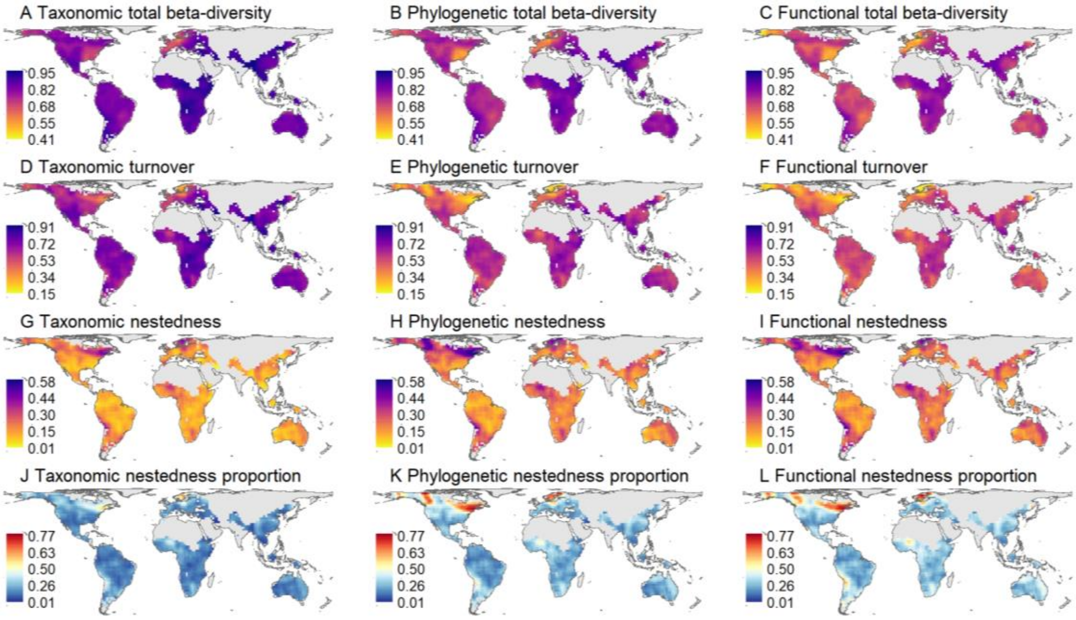

Fig. S14. Global patterns of taxonomic, phylogenetic and functional beta-diversity calculated with alpha-hull ranges using the alpha parameter of 2 for angiosperm trees. Total beta-diversity (**A**-**C**) and its components of turnover (**D**-**F**) and nestedness (**G**-**I**) and the proportion of total beta-diversity contributed by nestedness (**J**-**L**) are shown for three biodiversity facets, respectively. In **J**-**L**, the grid-cells with more than 50% of total beta-diversity contributed by nestedness are shown in red.

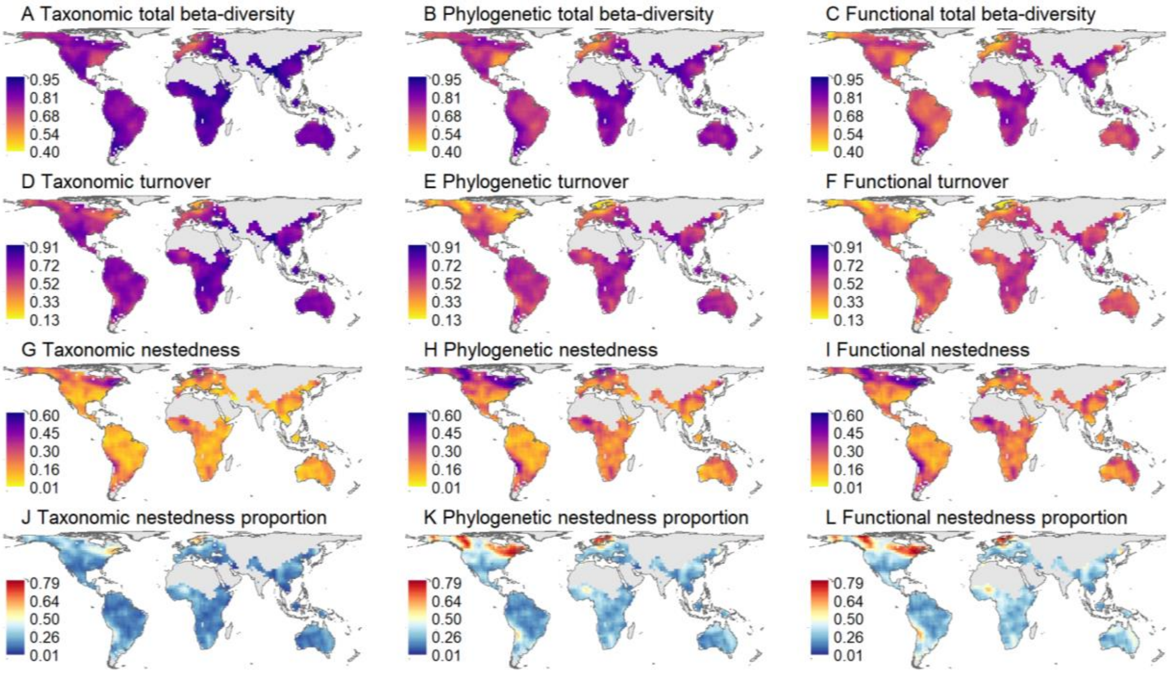

Fig. S15. Global patterns of taxonomic, phylogenetic and functional beta-diversity calculated with alpha-hull ranges using the alpha parameter of 4 for angiosperm trees. Total beta-diversity (**A**-**C**) and its components of turnover (**D**-**F**) and nestedness (**G**-**I**) and the proportion of total beta-diversity contributed by nestedness (**J**-**L**) are shown for three biodiversity facets, respectively. In **J**-**L**, the grid-cells with more than 50% of total beta-diversity contributed by nestedness are shown in red.

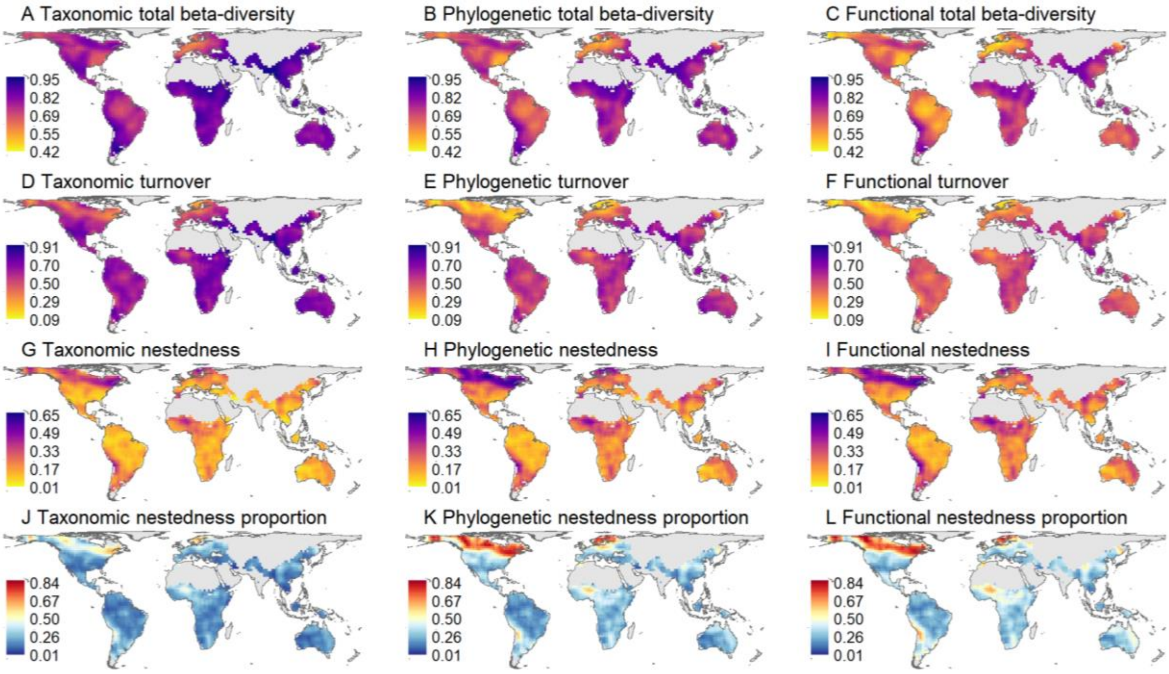

Fig. S16. Global patterns of taxonomic, phylogenetic and functional beta-diversity calculated with alpha-hull ranges using the alpha parameter of 10 for angiosperm trees. Total beta-diversity (**A**-**C**) and its components of turnover (**D**-**F**) and nestedness (**G**-**I**) and the proportion of total beta-diversity contributed by nestedness (**J**-**L**) are shown for three biodiversity facets, respectively. In **J**-**L**, the grid-cells with more than 50% of total beta-diversity contributed by nestedness are shown in red.

	A Alpha 2 versus Alpha 6			B Alpha 4 versus Alpha 6			C Alpha 10 versus Alpha 6		
	Taxonommic	Phylogenetic	Functional	Taxonommic	Phylogenetic	Functional	Taxonommic	Phylogenetic	Functional
Total	0.96	0.96	0.95	0.99	0.99	0.99	0.96	0.95	0.96
Turnover	0.97	0.97	0.97	0.99	0.99	0.99	0.97	0.96	0.96
Nestedness	0.96	0.97	0.97	0.98	0.99	0.99	0.97	0.97	0.98
Nestedness proportion	0.96	0.97	0.97	0.98	0.99	0.98	0.97	0.97	0.97

Fig. S17. Pearson's correlation coefficients between beta-diversity calculated with alpha-hull ranges using different values of the alpha parameter. The beta-diversity using the alpha parameter of 6 was compared to beta-diversity using the alpha parameter of 2 (**A**), 4 (**B**) and 10 (**C**). The comparisons were performed for total taxonomic, phylogenetic and functional beta-diversity and their respective components of turnover and nestedness and the proportion of total beta-diversity contributed by nestedness, respectively.

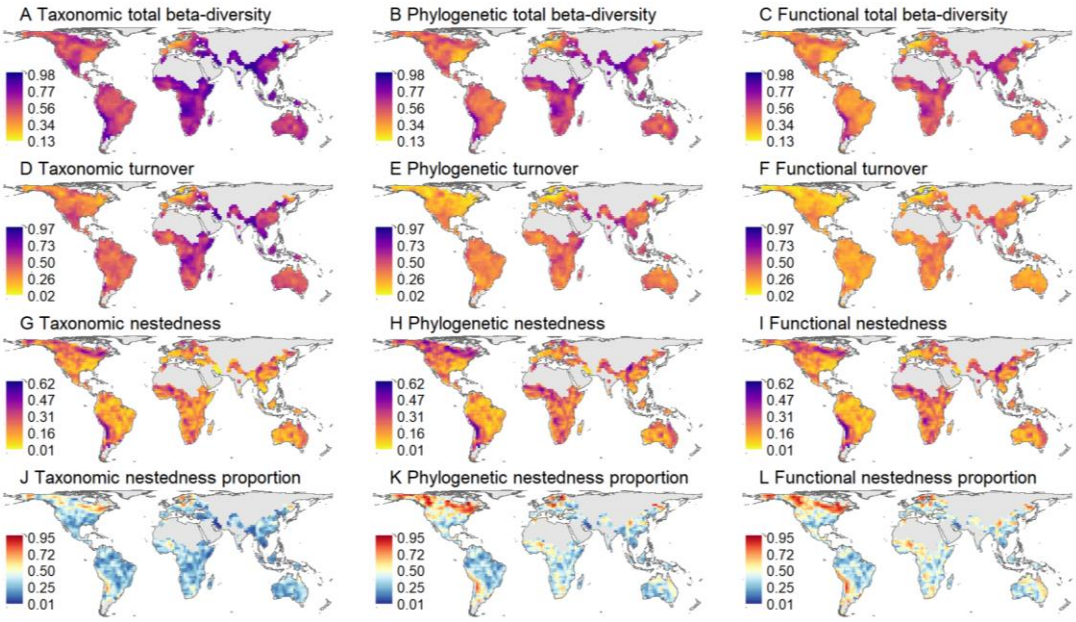

Fig. S18. Global patterns of taxonomic, phylogenetic and functional beta-diversity within moving windows of 9 (3×3) grid-cells for angiosperm trees. Total beta-diversity (**A**-**C**) and its components of turnover (**D**-**F**) and nestedness (**G**-**I**) and the proportion of total beta-diversity contributed by nestedness (**J**-**L**) are shown for three biodiversity facets, respectively. In **J**-**L**, the grid-cells with more than 50% of total beta-diversity contributed by nestedness are shown in red.

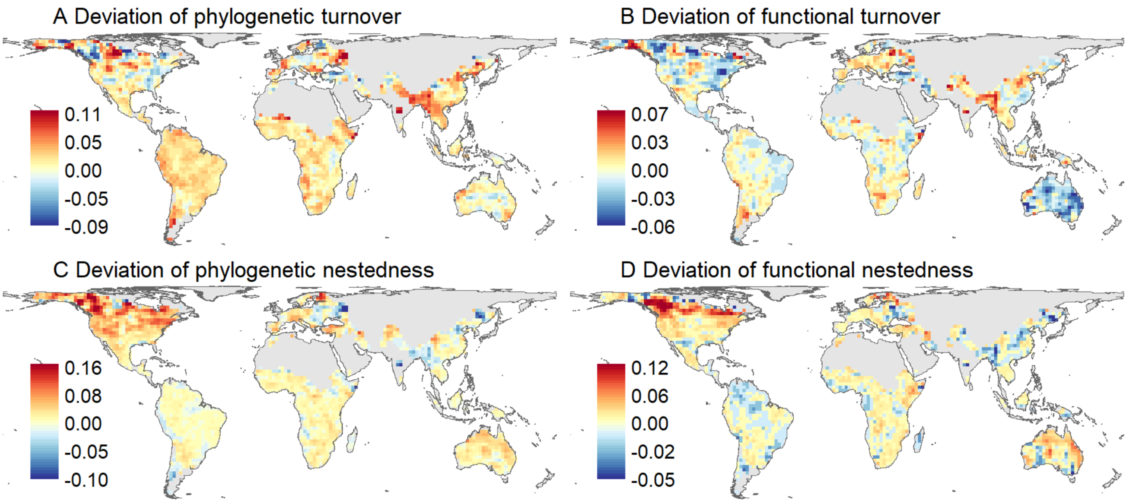

Fig. S19. Global patterns of the deviation of phylogenetic and functional turnover and nestedness within moving windows of 9 (3×3) grid-cells for angiosperm trees. The deviations of phylogenetic and functional turnover (**A, B**) and nestedness (**C, D**) were calculated as the differences between the observed and random expectations based on taxonomic beta-diversity and site-specific regional species pools.

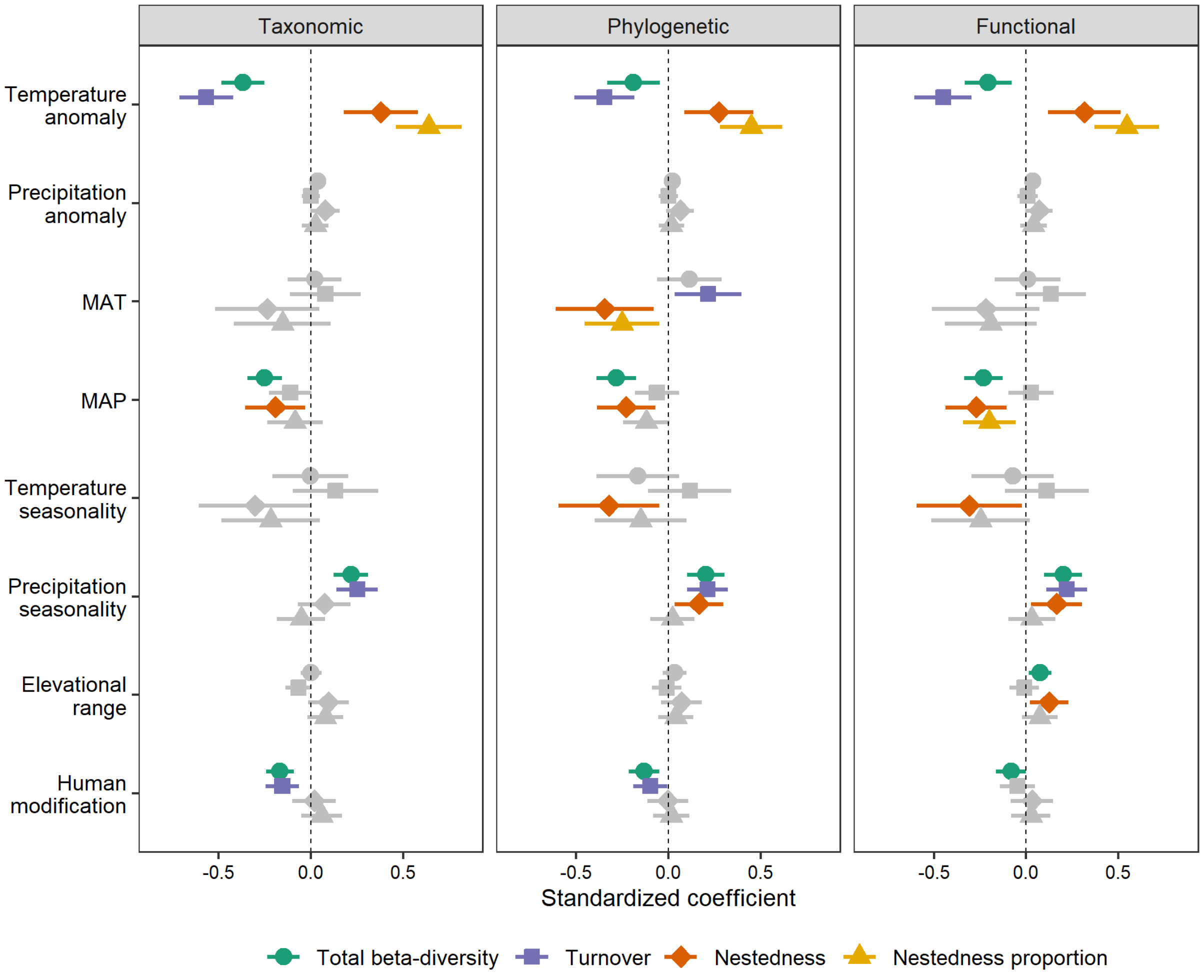

Fig. S20. Effects of past climate stability and current environmental conditions on taxonomic, phylogenetic and functional beta-diversity within moving windows of 9 (3×3) grid-cells for angiosperm trees. Total beta-diversity and its components of turnover and nestedness and the nestedness proportion were indicated with different shapes and colors. The averaged estimates of standardized coefficients (points) and the 95% confidence intervals (bars) were obtained from spatial simultaneous autoregressive models. Non-significant variables are shown in gray. Temperate and precipitation anomaly: the differences in annual temperature and precipitation between the present and the Last Glacial Maximum; MAT: mean annual temperature; MAP: mean annual precipitation.

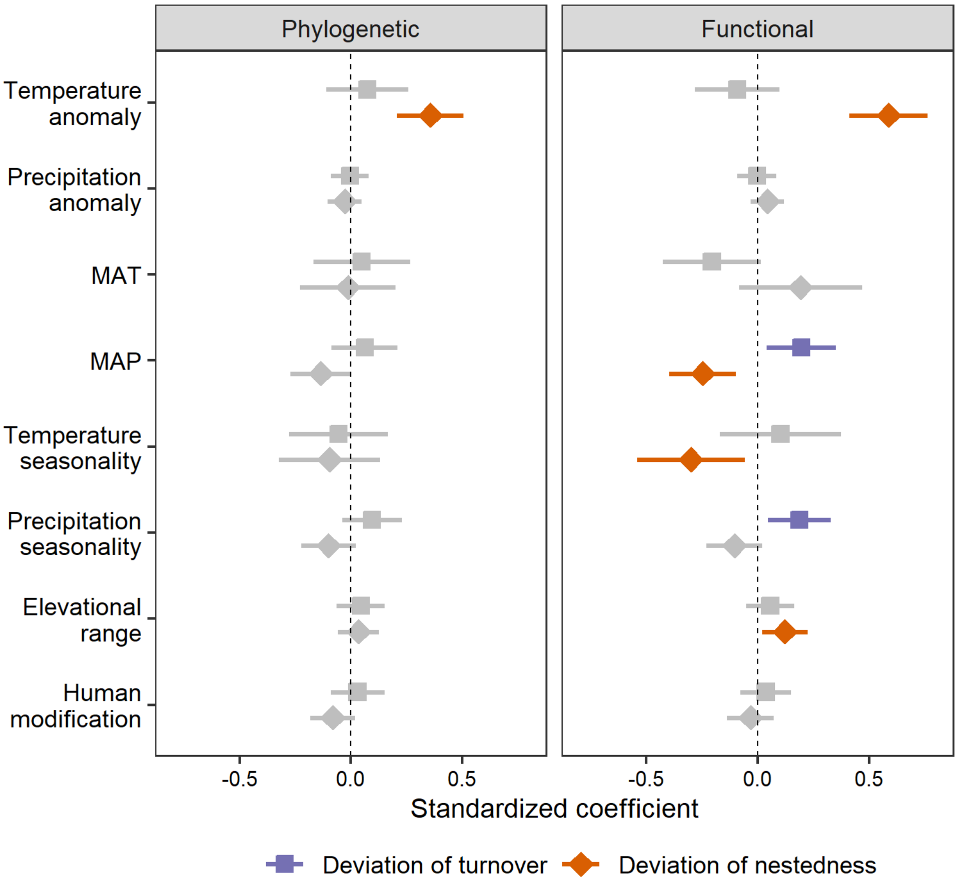

Fig. S21. Effects of past climate stability and current environmental conditions on deviations of phylogenetic and functional turnover and nestedness within moving windows of 9 (3×3) grid-cells for angiosperm trees. The deviations were calculated as the differences between the observed and random expectations based on taxonomic beta-diversity and site-specific regional species pools. The averaged estimates of standardized coefficients (points) and the 95% confidence intervals (bars) were obtained from spatial simultaneous autoregressive models. Non-significant variables are shown in gray. Temperate and precipitation anomaly: the differences in annual temperature and precipitation between the present and the Last Glacial Maximum; MAT: mean annual temperature; MAP: mean annual precipitation.

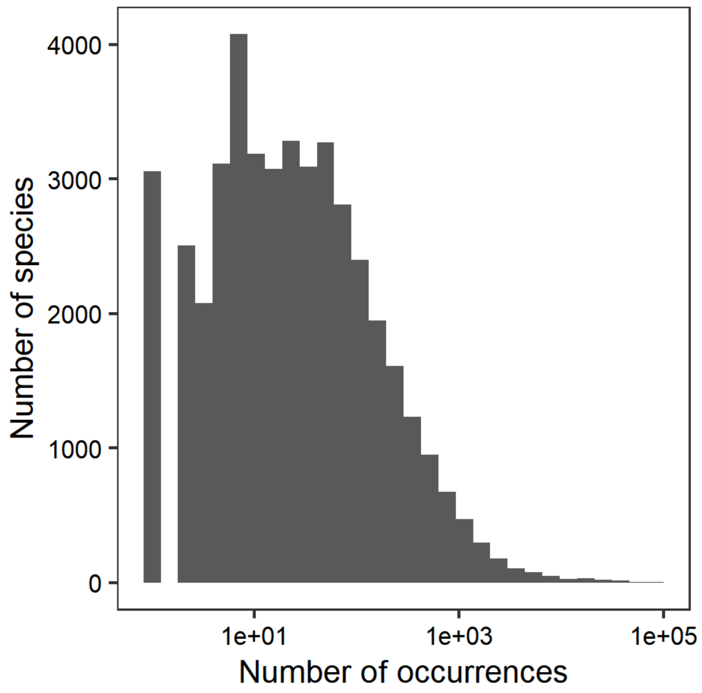

Fig. S22. Histogram of the number of occurrences for each angiosperm tree species that were used to estimate species ranges.

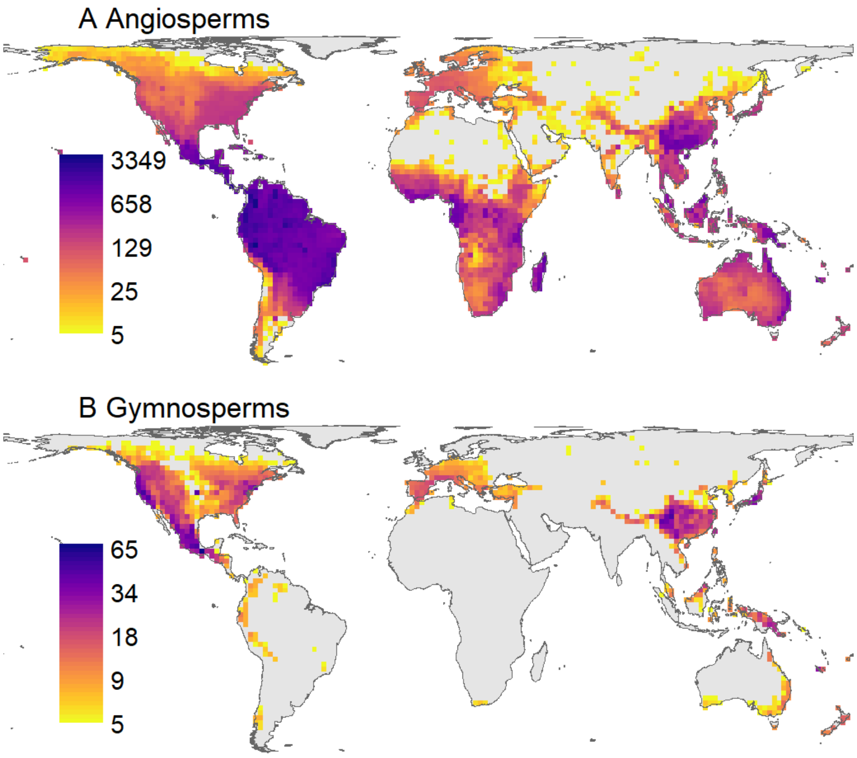

Fig. S23. Global pattern of species richness for angiosperm and gymnosperm trees. The grid cells with no data or fewer than five species are shown in gray.

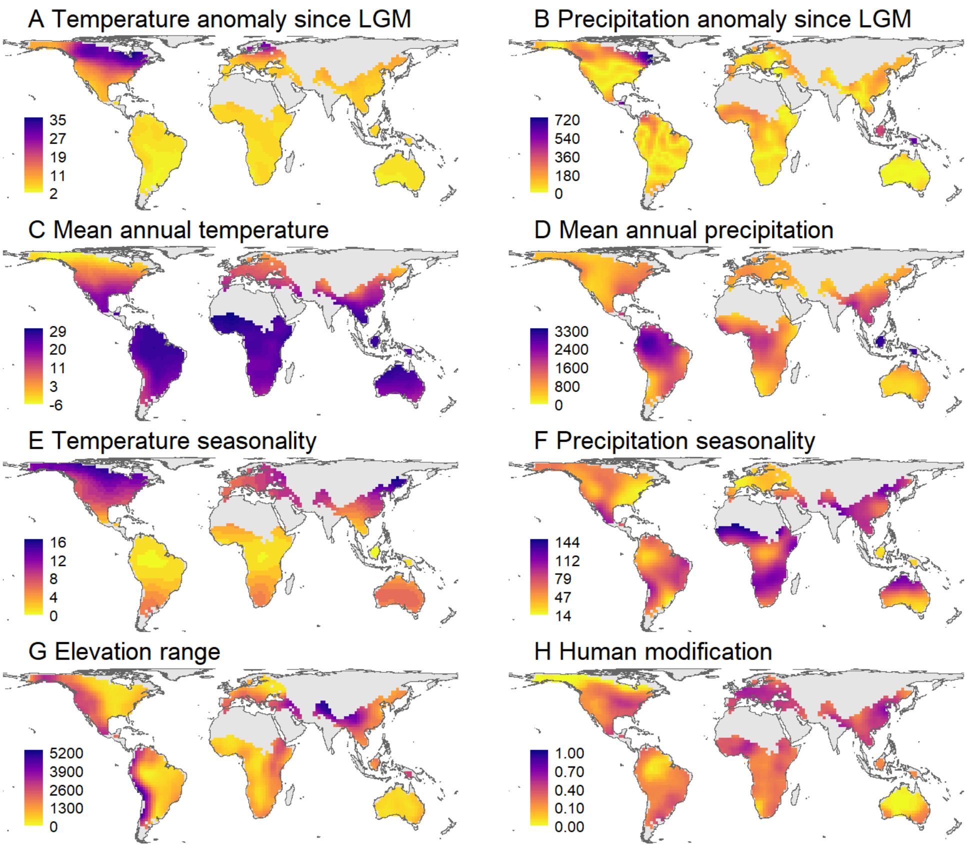

Fig. S24. Geographic patterns of eight environmental variables used in this study. To keep a consistent spatial extent of patterns of beta-diversity, only grid-cells with beta-diversity values were shown. Values of raw environmental conditions were averaged within moving windows of 25 (5×5) grid-cells of 200×200 km^2.

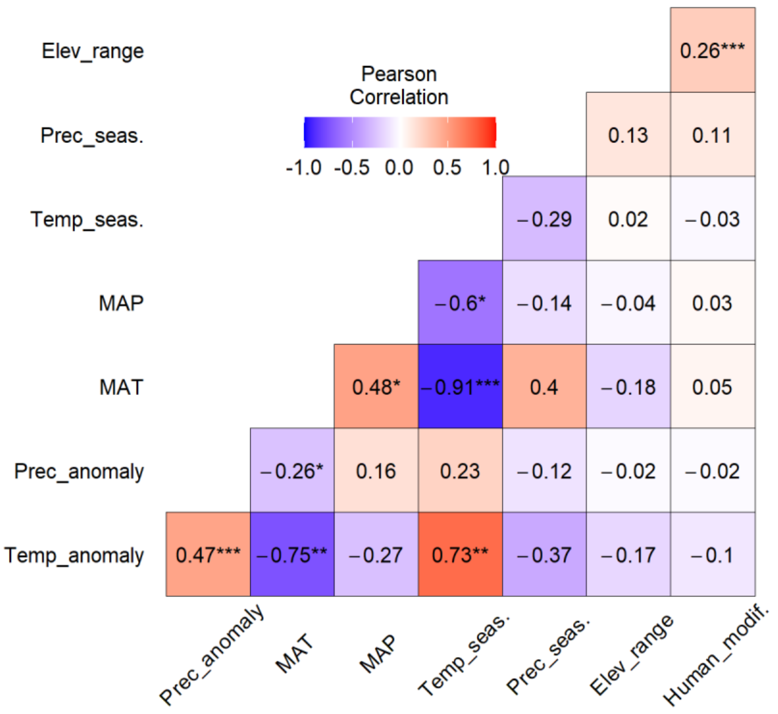

Fig. S25. Pearson's correlation coefficients among eight environmental variables. Significance was tested using a modified *t*-test to control for spatial autocorrelation. Temp_anomaly: temperature anomaly since the Last Glacial Maximum; Precp_anomaly: precipitation anomaly since the Last Glacial Maximum; MAT: mean annual temperature; MAP: mean annual precipitation; Temp_seas.: temperature seasonality; Prec_seas.: precipitation seasonality; Elev_range: elevational range; Human_modif.: Human modification index. *: $P < 0.05$; **: $P < 0.01$; ***: $P < 0.001$.

Table S1. Pearson's correlation coefficients between taxonomic, phylogenetic and functional beta-diversity (β) and eight explanatory variables for angiosperm trees. Metrics of beta-diversity included total beta-diversity and its components of turnover and nestedness and the nestedness proportion for three biodiversity facets, and deviations of phylogenetic and functional turnover and nestedness from random expectations based on taxonomic beta-diversity.

Facet	β metric	Temp. anomaly	Prec. anomaly	MAT	MAP	Temp. seas.	Prec. seas.	Elev. range	Human modif.
Taxonomic	Total dissimilarity	-0.28	0.05	0.26	-0.09	-0.18	0.55**	0.22	0.2
	Turnover	-0.6**	-0.32**	0.52*	0.14	-0.42	0.4*	0.2*	0.18
	Nestedness	0.58***	0.48***	-0.48***	-0.27	0.41**	-0.07	-0.08	-0.07
	Nestedness proportion	0.62***	0.46***	-0.53***	-0.26	0.45**	-0.16	-0.1	-0.1
Phylogenetic	Total dissimilarity	-0.21	0.1	0.21	-0.12	-0.16	0.53**	0.23	0.12
	Turnover	-0.71**	-0.34**	0.63**	0.24	-0.55*	0.4*	0.17	0.16
	Nestedness	0.69***	0.48***	-0.59**	-0.37*	0.53**	-0.08	-0.03	-0.11
	Nestedness proportion	0.74***	0.44***	-0.66**	-0.35	0.58**	-0.2	-0.06	-0.12
Functional	Total dissimilarity	-0.14	0.13	0.16	-0.14	-0.13	0.53**	0.25*	0.27
	Turnover	-0.65**	-0.33**	0.57**	0.22	-0.5*	0.38*	0.23*	0.29
	Nestedness	0.57***	0.47***	-0.47**	-0.35*	0.42**	0.05	-0.03	-0.08
	Nestedness proportion	0.66***	0.45***	-0.58***	-0.35*	0.52**	-0.1	-0.08	-0.17
Phylogenetic	Deviation of turnover	-0.47**	-0.19*	0.27	0.21	-0.31	0.24	0.16	0.13
	Deviation of nestedness	0.66**	0.26*	-0.5*	-0.28	0.49	-0.31	-0.09	-0.27
Functional	Deviation of turnover	-0.36*	-0.07	0.19	0.27	-0.29	0.17	0.3**	0.38
	Deviation of nestedness	0.63**	0.27**	-0.44*	-0.35	0.45	-0.2	-0.17	-0.35

Significance was tested using a modified *t*-test to control for spatial autocorrelation. Temp. anomaly: temperature anomaly since the Last Glacial Maximum; Precp. anomaly: precipitation anomaly since the Last Glacial Maximum; MAT: mean annual temperature; MAP: mean annual precipitation; Temp. seas.: temperature seasonality; Prec. seas.: precipitation seasonality; Elev. range: elevational range; Human_modif.: Human modification index. *: $P < 0.05$; **: $P < 0.01$; ***: $P < 0.001$.

Table S2. The 20 functional traits compiled that were used to fill data gaps for species missing trait values. Trait imputation was based on the correlation structure among traits, taxonomic hierarchy and phylogenetic information. Number of species, genera and families with trait data and their proportion of a total of 54,020 species, 4,157 genera and 253 families are reported for each functional trait. “All” means the number and proportion of taxa that have data for at least one functional trait. After imputation, eight traits were selected to calculate functional beta-diversity

Trait name	No. species	No. genera	No. families	% species	% genera	% families	Selected
All	11659	2318	225	21.6	55.8	88.9	
Wood density	6414	1582	188	11.9	38.1	74.3	√
Seed dry mass	4673	1358	195	8.7	32.7	77.1	√
Plant maximum height	4499	1270	187	8.3	30.6	73.9	√
Leaf N concentration	3579	1123	165	6.6	27	65.2	√
Leaf P concentration	2368	870	151	4.4	20.9	59.7	√
SLA	1965	725	138	3.6	17.4	54.5	√
Leaf N:P	1454	645	133	2.7	15.5	52.6	
Leaf K concentration	1242	559	126	2.3	13.4	49.8	
LDMC	793	396	102	1.5	9.5	40.3	√
LA	799	370	94	1.5	8.9	37.2	√
Leaf photosynthesis rate	768	415	116	1.4	10	45.8	
Seed germination rata	681	313	81	1.3	7.5	32	
LA (compund leaf)	665	312	86	1.2	7.5	34	
Stomatal conductance	478	295	95	0.9	7.1	37.5	
SLA_{FM}	281	185	68	0.5	4.5	26.9	
Leaf respiration rate	218	146	60	0.4	3.5	23.7	
LWR	192	108	50	0.4	2.6	19.8	
Fine root DM	72	60	33	0.1	1.4	13	
Leaf WUE	50	31	22	0.1	0.7	8.7	
Wood N	31	25	16	0.1	0.6	6.3	

N: nitrogen; P: phosphorus; K: potassium; SLA: specific leaf area; LDMC: leaf dry matter content; LA: leaf area (in case of compound leaves: leaflet, petiole and rachis excluded); LA (compound leaf): leaf area (in case of compound leaves: leaf, petiole included); SLA_{FM}: leaf area per leaf fresh mass (SLA based on leaf fresh mass); LWR: leaf dry mass per plant dry mass (leaf weight ratio); Fine root DM: fine root dry mass to leaf dry mass ratio; Leaf WUE: leaf photosynthetic water use efficiency; Wood N: wood nitrogen content per wood dry mass.

Table S3. Phylogenetic signals estimated with Pagel's λ for the eight traits used in this study. The phylogenetic signals were calculated for both measured and imputed trait data. For the imputed trait data, mean and sd (values in parenthesis) of phylogenetic signals were calculated for 200 random samples keeping the same number of species with measured data for each trait. All measured and imputed traits have significant phylogenetic signals ($P < 0.001$).

Trait name	Pagel's λ	
	Measured	Imputed
Wood density	0.943	0.985 (0.003)
Seed dry mass	0.954	0.989 (0.022)
Plant maximum height	0.855	0.974 (0.012)
Leaf N concentration	0.810	0.975 (0.006)
Leaf P concentration	0.796	0.974 (0.011)
SLA	0.847	0.978 (0.006)
LDMC	0.833	0.975 (0.018)
LA	0.602	0.982 (0.012)

N: nitrogen; P: phosphorus; SLA: specific leaf area; LDMC: leaf dry matter content; LA: leaf area.

Table S4. Summary results for full SAR models explaining taxonomic, phylogenetic and functional beta-diversity of angiosperm trees. Metrics of beta-diversity included total beta-diversity and its components of turnover and nestedness and the nestedness proportion for three biodiversity facets, and deviations of phylogenetic and functional turnover and nestedness from random expectations based on taxonomic beta-diversity.

Facet	β metric	Distance	minRSA	Max I	R^2
Taxonomic	Total dissimilarity	300	0.39	0.21	0.98
	Turnover	300	0.48	0.21	0.96
	Nestedness	300	0.56	0.25	0.93
	Nestedness proportion	300	0.55	0.24	0.94
Phylogenetic	Total dissimilarity	300	0.4	0.23	0.98
	Turnover	300	0.45	0.2	0.96
	Nestedness	300	0.6	0.26	0.94
	Nestedness proportion	300	0.53	0.23	0.95
Functional	Total dissimilarity	300	0.48	0.24	0.98
	Turnover	300	0.48	0.24	0.96
	Nestedness	300	0.61	0.3	0.94
	Nestedness proportion	300	0.54	0.27	0.94
Phylogenetic	Deviation of turnover	300	0.54	0.15	0.87
	Deviation of nestedness	300	0.59	0.19	0.92
Functional	Deviation of turnover	300	0.5	0.19	0.88
	Deviation of nestedness	300	0.52	0.18	0.92

Distance: radius (km) used to define neighborhood matrix; minRSA: minimum residual spatial autocorrelation (summed absolute values of Moran's I of first 20 distance classes); Max I: maximum Moran's I value in the first 20 distance classes; R^2: pseudo-R^2, squared Pearson correlation of predicted and observed values.
